# Deep Learning for Detecting and Locating Myocardial Infarction by Electrocardiogram: A Literature Review

**DOI:** 10.3389/fcvm.2022.860032

**Published:** 2022-03-25

**Authors:** Ping Xiong, Simon Ming-Yuen Lee, Ging Chan

**Affiliations:** ^1^State Key Laboratory of Quality Research in Chinese Medicine, Institute of Chinese Medical Sciences, University of Macau, Taipa, Macau SAR, China; ^2^Department of Public Health and Medicinal Administration, Faculty of Health Sciences, University of Macau, Taipa, Macau SAR, China

**Keywords:** deep learning, neural networks, electrocardiogram (ECG), myocardial infarction detection, myocardial infarction localization

## Abstract

Myocardial infarction is a common cardiovascular disorder caused by prolonged ischemia, and early diagnosis of myocardial infarction (MI) is critical for lifesaving. ECG is a simple and non-invasive approach in MI detection, localization, diagnosis, and prognosis. Population-based screening with ECG can detect MI early and help prevent it but this method is too labor-intensive and time-consuming to carry out in practice unless artificial intelligence (AI) would be able to reduce the workload. Recent advances in using deep learning (DL) for ECG screening might rekindle this hope. This review aims to take stock of 59 major DL studies applied to the ECG for MI detection and localization published in recent 5 years, covering convolutional neural network (CNN), long short-term memory (LSTM), convolutional recurrent neural network (CRNN), gated recurrent unit (GRU), residual neural network (ResNet), and autoencoder (AE). In this period, CNN obtained the best popularity in both MI detection and localization, and the highest performance has been obtained from CNN and ResNet model. The reported maximum accuracies of the six different methods are all beyond 97%. Considering the usage of different datasets and ECG leads, the network that trained on 12 leads ECG data of PTB database has obtained higher accuracy than that on smaller number leads data of other datasets. In addition, some limitations and challenges of the DL techniques are also discussed in this review.

## Introduction

According to the WHO's 2019 global health estimate, ischemic heart disease (IHD) has been the largest cause of death globally, accounting for 16% of deaths worldwide, and has increased from more than 2 million to 8.9 million in the last two decades (1). Myocardial ischemia is the first stage in the progression of myocardial infarction (MI) which is characterized pathologically as the irreversible necrolysis of cardiomyocytes caused by a disruption in coronary blood supply to the myocardium partially or completely (2). MI, also called a heart attack, can frequently occur in patients with a history of heart disease. Heart failure, angina pectoris, and arrhythmia are the main clinical symptoms of acute MI (3). Furthermore, studies reveal that ~22–64% of non-fatal MIs are silent or unrecognized MIs. Several potential factors, such as a history of cardiovascular diseases (CVD), hypertension, and diabetes could also increase the risk of silent MI, and this provides evidence that the prevalence and incidence of clinically unrecognized MI increase with patients' age (4, 5). The characteristics of asymptomatic and multifactorial may be related to a considerably increased mortality risk.

**Figure 1:**
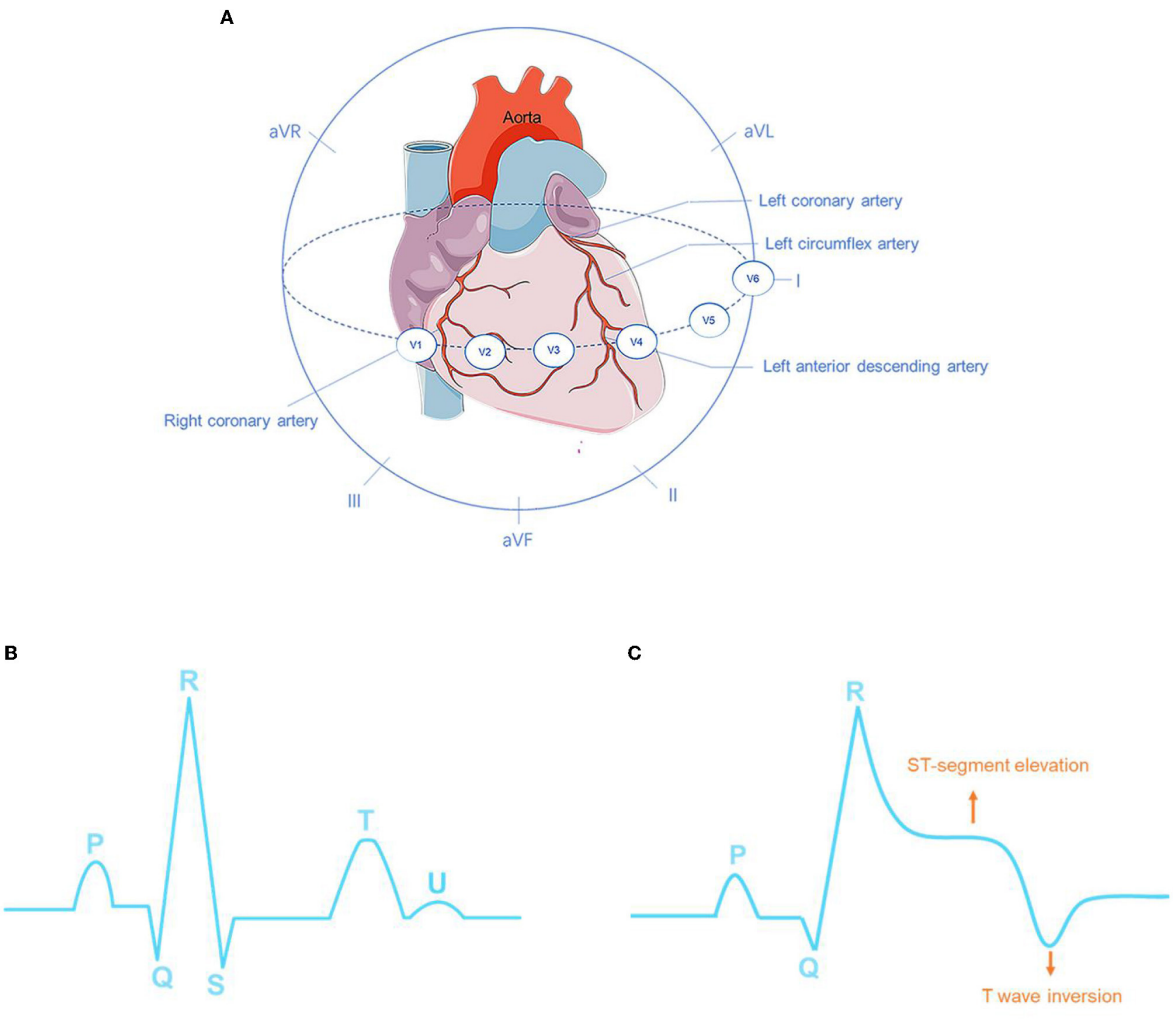
Overview of coronary arteries structure and ECG 12 leads **(A)**. The ECG characteristics of normal sinus rhythm **(B)** and myocardial infarction (MI) **(C)**.

Early diagnosis of the onset of MI is a crucial step for suspected patients to receive medical intervention timely, such as percutaneous coronary intervention (PCI) which is an effective way to limit infarct size and thereby reduce the risk of post-MI complications and heart failure (6, 7). Biomarkers, cardiac image modalities, and electrocardiographic methods play important roles in MI diagnosis (8). In terms of urgent therapeutic options, a non-invasive ECG is the most cost-effective and irreplaceable one and allows continuous and remote monitoring (9). The continuous ECG can always provide valuable prognostic information and may help determine the status of reperfusion or re-occlusion (10). Therefore, it is an essential diagnostic step for suspected patients either in pre-hospital or in-hospital settings. Additionally, the 12-lead ECG can be used to better understand the pathogenesis of MI and to pinpoint the localization of cardiac damage. Specific ECG leads can reflect the electrical activity of the heart from various angles, allowing them to distinguish between different types of MI based on the location of the infarction in the myocardium (11). For instance, a combination of lead V1, V2, V3, and V4 provides suggestive information concerning anterior MI (AMI), whereas a combination of lead II, III, and aVF can indicate inferior MI. However, the 12-lead ECG has difficulty in localizing the posterior MI (12, 13). Gupta et al. (14) quantified the contributions of each of 15 leads ECG signal from the PTB database individually and observed that the five leads: V5, V6, Vx, Vz, and II contain the most useful information, then they were quantified in pairs using the five best channels and results indicated that lead V6 and lead Vz can induce the best performance of the model. Fu et al. (15) employed an attention mechanism to select the most essential leads under the intra-patient and inter-patient scheme in MI detection and localization, although it just aimed to assist proposed DL methods to effectively diagnosis not to find the most significant leads in pathology. Treating all the leads equally could inversely lead to limiting model performance and largely increasing computational complexity because of redundant and unnecessary information. Multi-lead ECGs can not only help clinicians carry out myocardial reperfusion therapy as soon as possible but also help interventional cardiologists make targeted preliminary judgments on the pathological vessels related to infarction and perform directed interventional treatment. In addition, 12-lead ECG can also indicate whether clinicians need to prepare rescue measures for those patients with MI diagnosed with large infarct sizes. An overview of coronary arteries structure and ECG 12 leads is illustrated in Figure 1A. Different MI locations with their corresponding leads and culprit coronary arteries can be found in Table 1.

**Table 1:** Different myocardial infarction (MI) localizations and corresponding leads and culprit coronary arteries.

**MI locations**	**ST elevation**	**Reciprocal ST depression**	**T wave or Q wave**	**Culprit coronary arteries**
Antero-Lateral (ALM)	V3 ~ V6, I, aVL	None	None	LAD
Anterior (AMI)	V1 ~ V6	III and aVF	Hyperacute T waves Subsequent Q wave formation in precordial leads V1-V6	LAD
Antero-Septal (ASMI)	V1, V2, V3, or V4	None	Q waves in V1–V3 precordial leads	LAD
Septal	V1 and V2	None	None	LAD-Septal branches
Lateral (LMI)	I, aVL,V5, and V6	II,III, aVF	None	LAD and LCx
Inferior (IMI)	II, III, aVF	I, aVL (sensitive marker)	Hyperacute T waves Progressive development of Q waves in lead II, III, aVF	RCA (80%) or RCx (20%)
Posterior (PMI)	Require the extra leads V7–V9 (10). PMI accompanies 15–20% of STEMIs, the reciprocal changes of STEMI are sought in leads V1–V3 (16)	High R in V1–V3 with ST depression V1–V3 > 2 mm (mirror view)	Terminal T-wave inversion becomes an upright T wave	RCA or LCx

*LAD, left anterior descending coronary artery; RCA, right coronary artery; RCx or RCX, ramus circumflex artery; LCx or LCX, left circumflex artery*.

The main manifestations of MI in ECG are ST-segment elevation, as well as the high apex and inversion of T waves, and the appearance of pathological Q waves (Figure 1C). Among these manifestations, the change in the ST segment is the most significant. The characteristics of normal sinus rhythm and MI are shown in Figures 1B,C respectively. In a feature visualization analysis (17), the weight assigned to the U-wave of ECG signals is also large. It is also indicated that the U wave may play an important role in MI detection. Furthermore, silent MI with a Q wave accounted for 9–37% of all non-fatal MI events (18). According to ECG waveform characteristics, that is, whether ECG presents a specific sign called ST-elevation, MI can be divided into two categories: ST-elevation MI (STEMI), which refers to MI with ST-segment elevation that cannot be rapidly reversed by nitrates, and up to 25% of acute coronary syndrome (ACS) patients present this severe condition. Patients with STEMI will be at significant risk of cardiac mortality and sequelae if urgent reperfusion therapy is not provided (19). Non-ST-elevation MI (NSTEMI) is defined as MI with an ECG presentation of ST-segment depression, T-wave inversion, or both. The ECG waveform deviations provide indicative information about patients with MI. Pre-clinical ECG diagnosis can predict the risk stratification of MI and shorten the time to treatment. Patients with suspected STEMI who receive pre-hospital ECG have a 20% lower risk of in-hospital mortality (20). The interval between diagnosis and treatment is critical, and 12-lead ECGs of patients with MI should be collected and examined within 10 min of the initial medical contact (10). Silent patients with MI who receive treatment within 90 min of starting MI have a better chance of survival. In addition to being inefficient, manually identifying complicated non-linear ECG features across the 12-lead is time-consuming, and human interpretation of the ECG differs considerably depending on experience and competence levels. Because of intra- and inter-individual variability, neither timeliness nor accuracy can be guaranteed. It should be emphasized that the sensitivity and specificity of manual AMI diagnosis are 91 and 51%, respectively (21). As a result, prompt identification, immediate feedback, and precise diagnosis can give a better chance for future medical therapy.

## Machine Learning for MI Diagnosis

### Machine Learning

Machine learning (ML) is the technique of enabling computers to mimic human learning behaviors to update their existing knowledge frame and acquire new knowledge to progressively advance their ability to complete specific tasks (22). In cardiology, ML methods have been extensively used in medical imaging [e.g., CT (23), MRI (24), chest X-ray (25), echocardiogram (26)] and ECG (27). It is especially beneficial in pre-hospital settings, where paramedics may lack the knowledge of emergency physicians or cardiologists when it comes to interpreting ECGs. ECG interpretation using ML, including traditional ML, deep learning (DL), and a combination of the two. For traditional ML methods, extra hand-crafted feature extraction and selection steps are needed. Morphological features are computed by the demarcation of major ECG characteristic points such as the QRS complex, T wave, and the J point (28). Wavelet transforms decompose ECG signals in the time-frequency domain. Principal component analysis (PCA) (29), empirical mode decomposition (EMD) (30), and the hidden Markov model (31) are all commonly employed to extract representative features. The goal of feature selection is to lower the complexity of the computational process and ensure that ML algorithms only employ the most informative features. Conventional threshold-based support vector machines (SVM), random forest (RF), naive Bayes, decision tree (DT), k-nearest neighbor (KNN), and neural network (NN) are the commonly used classifiers, and almost all these techniques have achieved good performance beyond that of cardiologists, who achieved an average accuracy of 75% for detecting ECG pathologies (32). To name a few, Kora (33) proposed a hybrid firefly (FF) and PSO (FFPSO) algorithm to optimize ECG features. Then, an ANN model named the Levenberg–Marquardt Neural Network with optimization algorithm achieved the best accuracy of 99.3% compared with the other two ML algorithms, KNN and SVM. Acharya et al. (34) investigated a four-level discrete wavelet transform to extract 12 types of non-linear features. An ANOVA analysis was used to rank these features and obtain optimal features. The performance of the proposed model was evaluated using a KNN classifier, which had an accuracy of 98.8% for MI detection and 98.73% for MI localization. Some recent conventional ML methods with good performance for MI classifications are shown in Table 2. However, these ML techniques are also confronted with some challenges, which are concluded as follows:

**Table 2:** Recent conventional machine learning methods for MI detection and localization.

**References**	**Year**	**Feature extraction** **methods**	**Classification models**	**Number of hand-crafted features**	**MI detection**			**MI localization**			
					**Acc (%)**	**Sen (%)**	**Spec (%)**	**Nc**	**Acc (%)**	**Sen (%)**	**Spec (%)**
(32)	2016	DWT	SVM	35	95.30	94.6	96.0	5	98.1		
(33)	2017	FFPSO	LMNN KNN SVM		99.3 92.17 96.7	99.97 92.35 94.45	98.7 93.9 95.89				
(34)	2016	DWT and DCT	KNN	47	98.80	99.45	96.27	10	98.74	99.55	99.16
(35)	2018	SWT and sample entropy	KNN		98.69	98.67	98.72				
			SVM		98.84	99.35	98.29				
(36)	2018	PCA Clinical features	SVM		96.66	96.66	96.66				
(37)	2019	FBSE-EWT	LSSVM	108	99.97	100	99.95				
(38)	2021		SVM	24 temporal, 288 morphological, 3 non-linear	97.00	97.33	96.67				
(39)	2020	DWT, PCA	NN	28 (detection) 32 (localization)	98.21	97.5	98.01	6	98.22	98.14	99.40
(40)	2012	Time-Domain	KNN	36		99.97	99.90	10		96.72	97.11

*DWT, Discrete wavelet transform; FFPSO, Hybrid Firefly and Particle Swarm Optimization algorithm; DCT, Discrete cosine transform; SWT, Stationary wavelet transform; PCA, Principal component analysis; FBSE-EWT, Fourier–Bessel series expansion-based empirical wavelet transform; LMNN, Levenberg Marquardt neural network; LSSVM, The least square-support vector machine; Acc, Accuracy; Sen, Sensitivity; Spec, Specificity; Nc, Number of classes*.

- First, information loss: the feature extraction and classification are two isolated modules in traditional ML approaches. In feature engineering, dimensionality reduction can remove large irrelevant features, make data analysis simpler, and lower computational costs. The feature selection aims to choose an appropriate algorithm to rank the scores of contributions of the features to the results so that the relevant characteristics can be maintained (41). However, the unselected features in the process of feature selection are directly moved out, so it is difficult to tell if the hidden information has been thoroughly unearthed or utilized redundantly. This form of two independent modules gives a negative influence on the learning ability and performance of ML models (42).
- Second, in the conventional ML framework, independent feature extraction techniques use fixed hand-crafted features. However, ECG characteristics may change with the influence of some external factors such as patients' age, gender, the devices used for acquiring ECG data, and the generalizability is compromised when fixed ECG features are used (43).
- Third, feature point detection cannot be guaranteed in ECG signals due to their faintness and noise interference. When confronted with faulty ECG tracings, traditional models can easily lose their robustness (41).
- Fourth, the ECG characteristics of MI, such as ST-segment deviation, are often inadequate to detect MI since they may be seen in other cardiac conditions, such as left ventricular hypertrophy and left bundle-branch block (44). Moreover, ECG abnormalities are also common in patients who have myocarditis or Takotsubo syndrome (TTS) (45, 46) Therefore, the low accuracy of the conventional ML methods keeps the manual-crafted feature extraction as a central task, which may be attributed to the traditional imperfect ECG classification criteria.

### Deep Learning

Deep learning (DL) is a subfield of ML. DL models can be trained on huge datasets to learn the relationship between the input features and the results automatically (47) Thus, they can directly learn features from given input of raw data without a specific step of feature extraction and have the capacity to maintain good generalizability. The hidden layers of DL models are a black box, which is responsible for automatic features learning. The deep hidden layers make a deep neural network that can potentially map to any function, which allows it to solve exceedingly complicated functions. A system with 5–20 non-linear layers may implement extraordinarily complex functions of its inputs (47). DL has been rapidly evolving and having an imperative impact on the accuracy in the classification of heart diseases. Data-driven DL models rely significantly on the quality of data and can be promoted as more data is gathered. Since 2010, due to aging populations, the availability of easy-to-use ECG monitoring devices in the form of wireless, mobile, and remote technologies have greatly expanded the capture of ECG data, and DL algorithm-based interpretation software automatically interprets ECG data. Wearable technology, wireless sensors, and deep learning techniques can all collaborate to create innovative approaches to improve healthcare services (48, 49). The improvement of AI may greatly promote the development of ECG and expand the interoperability of healthcare. For the limitations of conventional ML approaches mentioned in Section Machine Learning, the advantages of DL methods compared with the ML approaches are concluded as follows:

- As opposed to the conventional ML algorithms, the DL framework integrates feature extraction and classification into a whole instead of clearly describing them as two independent modules. That is also called the “end-to-end” model. End-to-end models can use a single model to solve tasks with multiple modules or steps. When solving a complex task using multiple modules, the major drawback is the accumulation of errors, since deviations from one module can affect the next. The end-to-end DL models avoid this inherent defect and make a reduction in engineering complexity. However, just as the “no Free Lunch” theory, the interpretability of DL models is reduced.
- From the perspective of generalization, DL is easier to deal with massive data than conventional ML methods. It is believed that memory training data is an important reason for the poor generalization ability of models in conventional view. Therefore, various regularization methods are often used to make models “simple” and break this memory. In Zhang et al. (50), researchers challenged this conventional view by adopting the randomization test to compare how the learning algorithm performs on the natural data vs. the randomized data. The results indicate that deep neural networks easily fit random labels and emphasize that the effective capacity of the neural network makes it large enough to memorize data. More importantly, researchers assessed the effects of implicit regularizers on generalization performance and proved implicit regularizations are not the main cause for model generalization. Therefore, we can infer deep neural networks make good use of their memory when they work. In comparison, the traditional ML methods lack this memory when they deal with complex functions.
- Compared with the rule-based features extraction, DL approaches automate feature engineering. DL models can better express the rich underlying information of data by using vast amounts of raw data to automatically identify the representations needed for classification. As a result, the DL algorithms may “see” informative features that even the experienced expert may not notice. Their potential for greater generalization ability makes them better adapt to the dynamic changes of ECG patterns and enables them to diagnose more cardiac conditions with a greater performance than identified by conventional ML.

**Figure 2:**
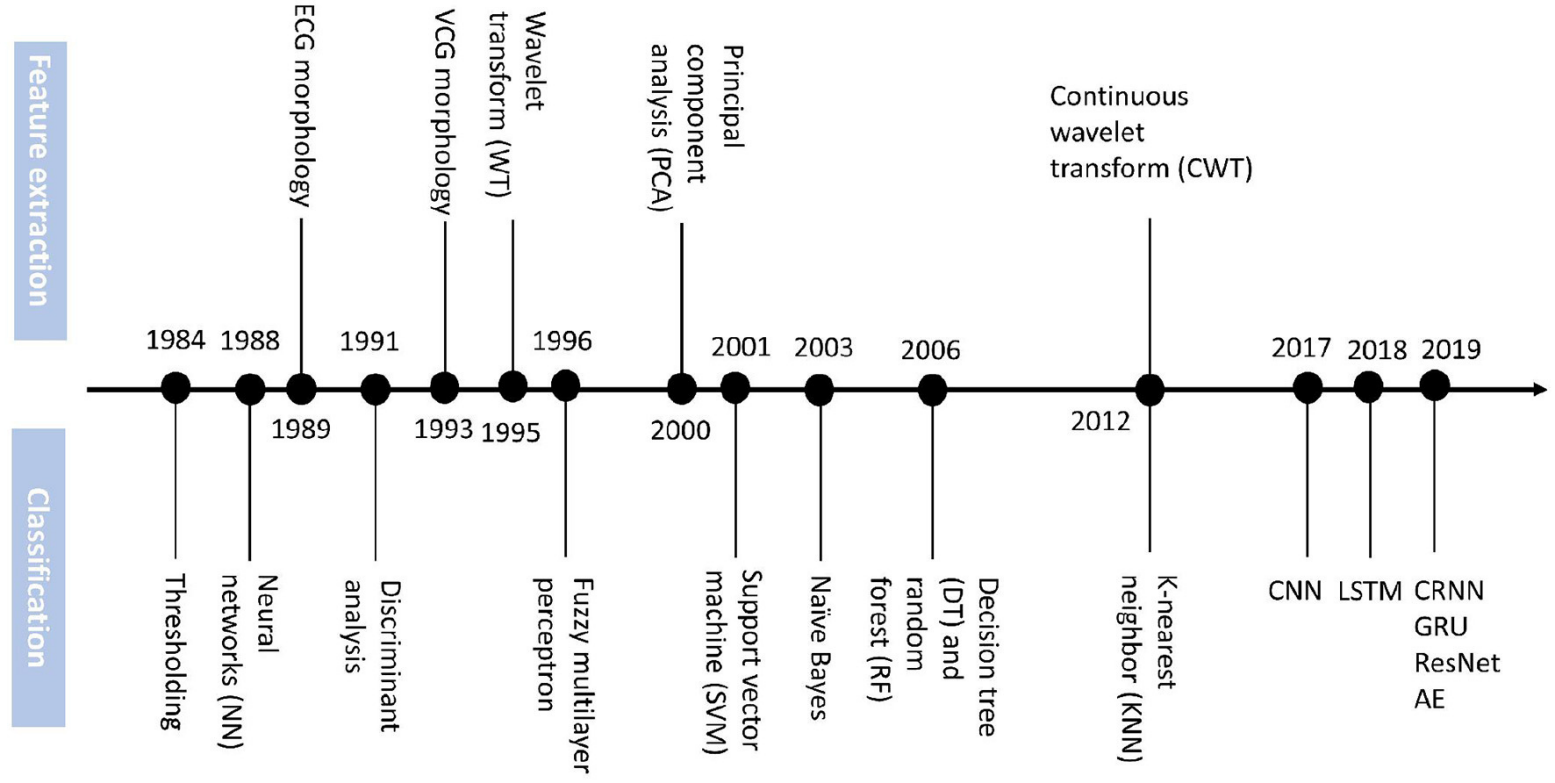
Timeline of technology advances of feature extraction and classification methods for MI diagnosis.

Based on the summary of Ansari et al. (51) we add the DL methods for MI detection and localization emerging from the recent 5 years to construct the timeline in Figure 2.

## Objectives and Methods

### Other Related Work

There exist other six related works that focus on automatic ECG analysis for the prediction of structural cardiac pathologies, including two systematic reviews (52, 53), one meta-analysis (54), and three comprehensive reviews (44, 51, 55). Al Hinai et al. (52) assess the evidence for DL-based analysis of resting ECGs to predict cardiac diseases such as left ventricular (LV) systolic dysfunction, myocardial hypertrophy, and ischemic heart disease. Joloudari et al. (53) focus on ML and DL techniques for myocardial infarction disease (MID) diagnosis but just cover 16 papers regarding DL methods. Grün et al. (54) include a total of five reports to provide an overview of the ability of AI to predict heart failure based on ECG signals. Attia et al. (55) discuss AI ECG algorithms for cardiac screening including LV dysfunction, silent atrial fibrillation, hypertrophic cardiomyopathy, and other structural and valvular diseases. Jothiramalingam et al. review papers that consider ECG signal pre-processing, feature extraction and selection, and classification techniques to diagnose heart disorders such as LV Hypertrophy, Bundle Branch Block, and MI. Ansari et al. (51) comprehensively evaluate several hundred publications that analyzed the ECG signal and electronic health records (EHR) to diagnose myocardial ischemia and infarction automatically and point out that DL methods have not specifically been used to detect MI and ischemia prior to 2017.

### Study Objectives

In recent 5 years, there has been an emerging number of studies focusing on state-of-the-art DL methods for MI detection and localization. Compared to the related work mentioned before, we only focus on DL techniques for MI detection and localization to consider more related papers. Covering a wide variety of studies focused on this topic in the review, we have set the following detailed goals: First, to describe and evaluate all public databases and newly collected datasets, as well as the commonly accepted assessment measures employed in the reviewed publications. Second, to assess and compare the different DL methods based on their respective performance in detecting and locating MI to find the most popular one. Finally, to discuss how these different DL methods contribute to MI detection and localization, as well as the primary potential obstacles of using DL algorithms in clinical practice for MI detection.

### Study Selection

**Figure 3:**
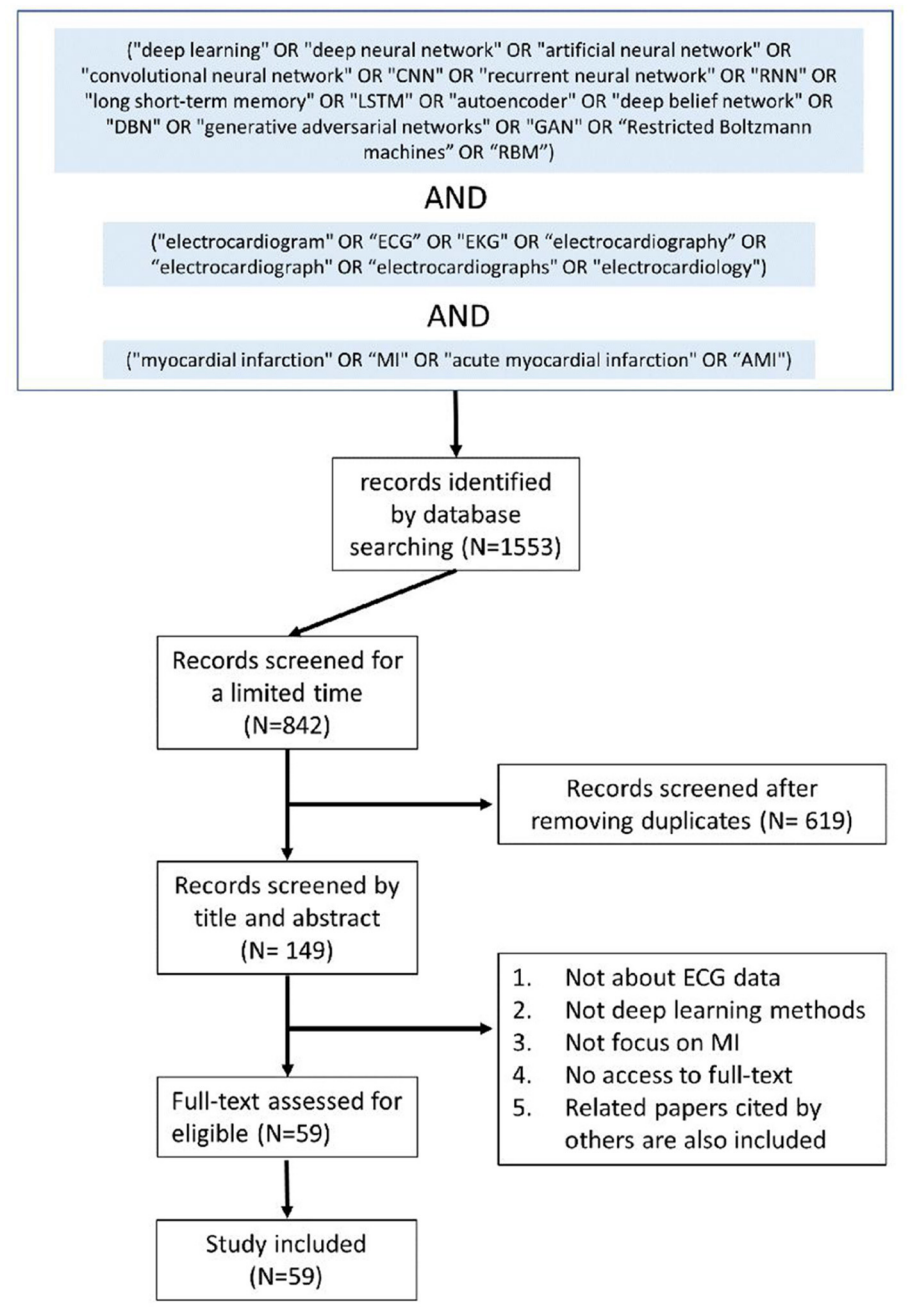
Flow diagram of paper selection.

We search technical papers that deployed DL methods [deep neural networks (DNNs)] for MI detection and localization on Google Scholar, PubMed, and Web of Science. The following general search terms are used: (“deep learning” OR “deep neural network” OR “artificial neural network” OR “convolutional neural network” OR “CNN” OR “recurrent neural network” OR “RNN” OR “long short-term memory” OR “LSTM” OR “autoencoder” OR “deep belief network” OR “DBN” OR “generative adversarial networks” OR “GAN” OR “Restricted Boltzmann machines” OR “RBM”) AND (“electrocardiogram” OR “ECG” OR “EKG” OR “electrocardiography” OR “electrocardiograph” OR “electrocardiology”) AND (“myocardial infarction” OR “MI” OR “acute myocardial infarction” OR “AMI”). To avoid missing related papers, we also include some references that have been cited by others. Figure 3 shows the flow diagram of paper selection.

## Findings From Review

### Datasets

A high-quality dataset can boost the improvement of data-driven model performance and generalizability, making DL techniques possible to be used in clinical environments. A total of 13 datasets are considered in all the reviewed papers. The largest database is the ECG-ViEW II database consisting of 979,273 recordings from 461,178 patients, which was collected over a 19-year study period in South Korea. The smallest dataset is the Long-Term ST Database (LTST), only covering 86 recordings from 80 subjects, which were collected in Slovenia. Thus, these datasets differ significantly in the number of ECG recordings. Among the investigated papers, 47 (more than 79%) research trained DL models on the public Physikalisch-Technische Bundesanstalt (PTB) Diagnostic ECG database and achieved high performance for MI classification. It makes the problem of the generalizability of these existing DL models worth considering, and clinical data is needed to verify the diagnostic efficacy of such models. Other public databases such as the LTST, the PTB-XL, and the ECG-ViEW II database are alternatives for some researchers. Notably, 7 studies are using the new ECG collections from some medical institutes in recent two years. For instance, Tadesse et al. (56) collected the ECGs from 17,381 patients (11,853 MI and 5,528 Normal cases) in the Provincial Key Laboratory of Coronary Heart Disease, Guangdong Cardiovascular Institute (GCI), and three subgroups: acute, recent, and old MI were divided by cardiologists based on patients' medical history with a combination of ECGs. In 12/13 of these datasets, ECGs are collected as voltage amplitude time-series signals in one dimension, however, there is also one ECG images collection for CNN model training. Khan et al. manually collected 11,148 ECG images from Ch. Pervaiz Elahi Institute of Cardiology Multan in Pakistan and cardiologists annotated the images. All collected datasets are listed in Table 3.

**Table 3A:** Properties of the collected databases used in the research of MI detection and location.

**Dataset**	**Country**	**Year**	**Np**	**ECG recordings information**	**Strength**	**Limitation**	**Link**
PTB (57)	Germany	2000	47	**15 leads:** 12 leads + 3 Frank leads (Vx, Vy, and Vz) **549 records from 290 subjects:** (Aged 17–87, mean 57.2; 209 men, mean age 55.5, and 81 women, mean age 61.6) **Length:** 2 min **Frequency:** 1 kHz (available up to 10 kHz) **Resolution:** 16 bits with 0.5 V/LSB (2,000 A/D units per mV)	15 leads ECG are included Higher resolution than LTST	Small sample size	https://physionet.org/content/ptbdb/1.0.0/ or https://doi.org/10.13026/C28C71
The LTST (58)	Slovenia	2003–2007	2	**2 or 3 leads** **86 recordings from 80 subjects:** 1,155 (ischemic), 335 (non-ischemic) ST episodes **Length:** 21–24 h **Frequency:** 250 Hz **Resolution:** 12-bit over a range of ±10 millivolts **Annotations:** locations of the PQ junction (the isoelectric level) and the J point, ST level time series or the ST deviation time series	All 86 data are supplied with detailed annotations and ST deviation trend plots	Data sample size is small Just 2 or 3 leads ECG are included	https://physionet.org/content/ltstdb/1.0.0/ or https://doi.org/10.13026/C2G01T
PTB-XL (59)		1989–1996	1	12-lead **21,837 recordings from 18,885 patients** (Male: Female = 52:48%) (Ages: from 0 to 95 years Median 62 and interquartile range of 22) **Length:** 10 s **Frequency:** 500 Hz	The to-date largest freely accessible clinical 12-lead ECG-waveform dataset		https://physionet.org/content/ptb-xl/1.0.1/
ESCDB (60)	The European Community	1985	1	Lead 3 (L3) and Lead 5 (L5) **90 annotated ECG recordings from 79 subjects** 367 episodes of ST segment change, and 401 episodes of T-wave change, with durations ranging from 30s to several minutes **Length:** 2 h **Frequency:** 250 Hz **Resolution:** 12-bit over a nominal 20 mv input range	Beat by beat annotations are included	Only 2 leads are included and small sample size Contains nonischemic ST-segment changes	https://www.physionet.org/content/edb/1.0.0/ or https://doi.org/10.13026/C2D59Z
ECG-ViEW II (61)	South Korea	1994–2013	1	**12 leads** **979,273 recordings from 461,178 patients** over a 19-year study period **ECG parameters:** QT interval, QTc interval, RR interval, PR interval, QRS duration, P wave axis, QRS axis, and T wave axis	Based on real-world clinical practice data of patients who have taken medicines to treat various diseases Consists of long-term follow-up data	The algorithms of calculating ECG, parameters were not upgraded in the period of collecting time Data are from one hospital No waveform data are provided	http://www.ecgview.org
STAFFIII (62)	USA	1995–1996	2	**12 leads** The database consists of 104 patients and a total of 152 occlusions in the major coronary arteries 35 patients had previous MI **Frequency: 1,000 Hz** **Resolution: 0.625** **μV**	It accounted for inter-patient variability in reaction to prolonged balloon inflation as well as variability of heart rhythm and waveform morphology		https://www.physionet.org/content/staffiii/1.0.0/ or https://doi.org/10.13026/C20P4H

*Np, number of papers*.

**Table 3B:** Description of newly collected datasets used in studied research.

**Dataset's name/description**	**Country**	**References**	**Properties of ECG data**
The ICBEB	China	(63)	825 records for ST-segment depression (STD), and 202 records for ST-segment elevated **Length:** 6–60 s **Frequency:** 500 Hz
A dataset built by Chapman University, Orange, CA, USA, and Shaoxing People's Hospital, China	USA and China	(64)	12-lead ECGs from 10,646 patients **Length:** 10 s **Frequency:** 500 Hz 11 common heart rhythms and 67 additional cardiovascular conditions, with the images labeled by cardiovascular experts
GGH	China	(63)	12-lead ECG from 21,241 anonymized patients 15,578 MI cases and 5, 663 normal cases **Frequency:** 500 Hz
The ECGs records which were collected in the Provincial Key Laboratory of Coronary Heart Disease, Guangdong Cardiovascular Institute (GCI)	China	(56)	The 12-lead ECGs from 17,381 patients (11,853 MI and 5,528 Normal cases) 1,489 Acute (MI occurred within 7 days), 5,377 Recent (MI occurred in <30 days but longer than 7 days) and 4,613 Old (MI occurred beyond 30 days) MI cases **Length:** 10 s **Frequency:** 500 Hz
Hospital A was a cardiovascular teaching hospital and hospital B was a community general hospital	South Korea	(65)	**12 leads** **Length:** 10 s **Frequency:** 500 Hz
A collection of 11,148 standard 12-lead-based ECG images were obtained from Ch. Pervaiz Elahi Institute of Cardiology Multan, Pakistan	Pakistan	(66)	2,880 images for MI 2,796 for abnormal heartbeats 2,064 for previous history of MI 3,408 for normal
114 patients enrolled in the Kerckhoff Biomarker Registry for the training and evaluation of the deep neural networks	Germany	(67)	The 12-lead ECG recordings from 114 patients **Length:** 10 s **Stored format**: XML-based HL7v3 ECG acquisition devices: Cardiovit AT-102P, Schiller-Reomed AG, Obfelden, Switzerland **Frequency:** 0.05–150 Hz; **Measuring range**: ±300 mV; **Sampling rate:** 500 data points per second/5,000 in 10 s (per lead); **Digital resolution:** 5 μV/18 bit

**Figure 4:**
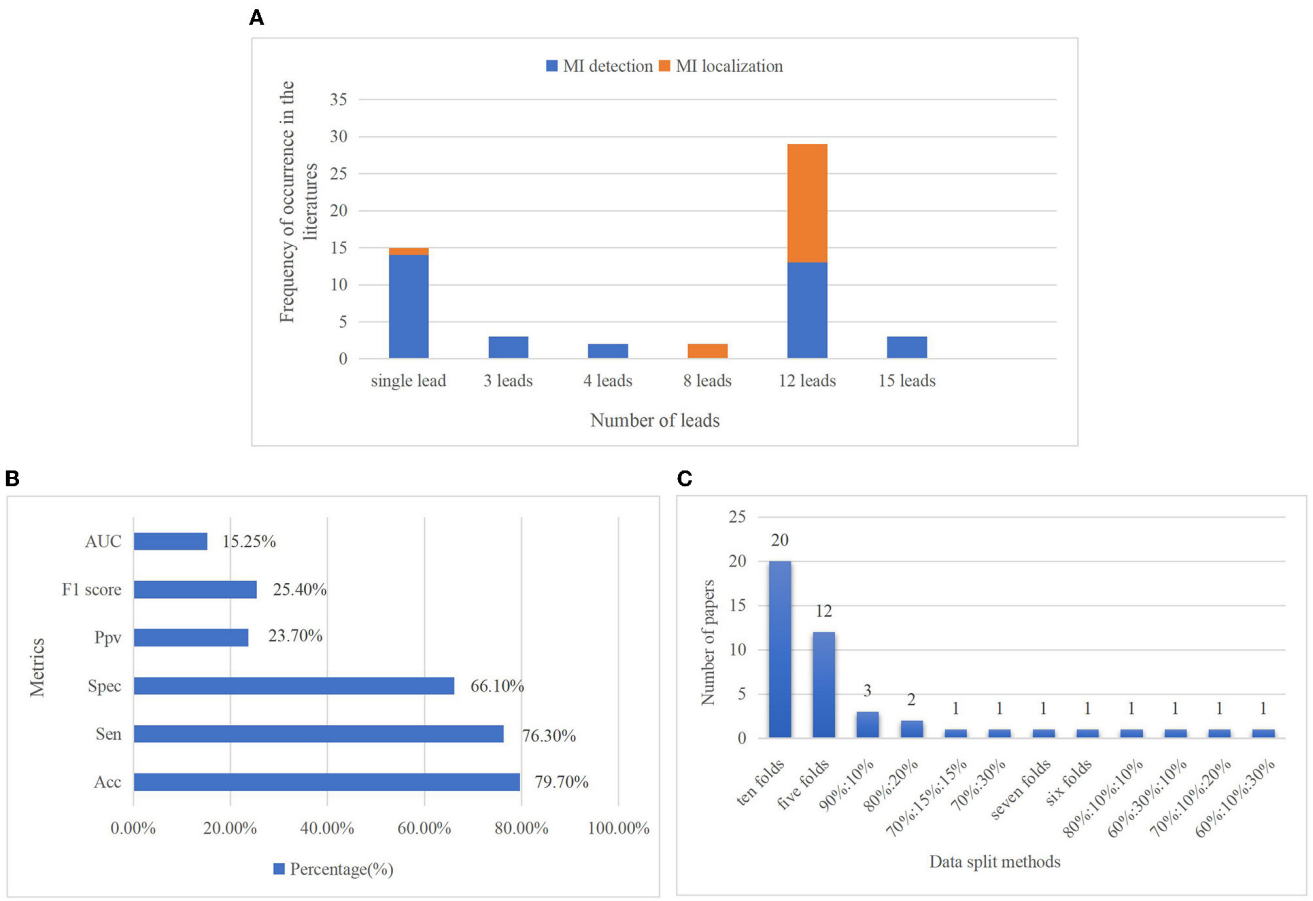
The number of leads used in investigated articles **(A)**. The proportion of the usage of single lead especially the lead II ECG signals is the highest for MI detection, even higher than the standard 12-lead ECG signals. It is because this limb lead is coaxial to the cardiac conduction, the ECG signal in lead II has the largest forward waveform amplitude and the clearest waveform amplitude, so it can provide good ECG morphological information for MI detection (74). Furthermore, for the objective of MI localization, only 12-lead ECG data can provide comprehensive information and reflect different regions of the heart, so standard 12 lead ECG signals are employed in most research of MI localization. Percentage of metrics used in reviewed papers **(B)**. Accuracy, sensitivity, and specificity are the three major metrics. Accuracy, which is considered in 79.7% of the articles, is the most frequently used metric, followed by sensitivity and specificity, with rates of 76.3 and 66.1%, respectively. Less than 20% of authors have considered AUC. Distribution of data splitting methods in MI diagnosis **(C)**. They contain k-fold cross-validation (CV) such as ten- and five-folds, train-test separation such as 90:10 and 80:20%, and train-validation-test separation such as 70:15:15 and 60:10:30%. It shows tenfold CV is the most popular data splitting method, which has been considered in 20 articles.

- Leads: Standard 12 leads ECG are included in ten datasets, but few datasets only contain two or three leads ECG recordings such as the LTST and The European ST-T database (ESCDB). Each record in the PTB database includes 12 conventional standard leads together with a vectorcardiogram (VCG) of three Frank leads, and the 15 leads ECG can provide a more comprehensive assessment about the heart abnormalities. It is also the only database whose record has 15 simultaneously measured signals in all the datasets. In the reviewed literature, a small number of them adopted ECG signals of several specific leads according to their different study objectives, for instance, Lead V1, V2, V3 are adopted to detect AMI (68), and lead II, III, and aVF are used for detection of IMI (69). The three VCG leads record electrical heart activities in three orthogonal planes including frontal, sagittal, and transverse (70). In Yadav et al. (71), the best results obtained for 3 VCG leads were concatenated. According to Einthoven's law, there are linear correlations between limb leads, and these leads are electrically equilateral (72). Therefore, 8 leads (2 limb lead and 6 precordial leads) are considered as the non-redundant number of leads in Zhang et al. (73) to generate model training. The usage of ECG leads is shown in Figure 4A.
- Length of ECG recordings: Several databases contain short-term ECG recordings which are less than several minutes, such as 10 s (59) and 2 min (57), while some databases include long-term ECG data which is more than 1 h, such as 2 h (60) and 24 h (58). Sometimes, the length of 2 h is also relatively short for MI detection and limits the use of computational methods that analyze the signals over a longer period.

### Model Performance Evaluation

#### Metrics

Model performance evaluation is the main step in ML-based diagnosis. There are plenty of measurements for the performance of various diagnostic algorithms, just like accuracy, sensitivity, specificity, precision, F1-score, receiver operating characteristic curve, and area under the curve. There is still a lack of consistency in the performance metrics used for MI classification through using robust evaluation strategies to make results comparable and generalizable is of great importance. Figure 4B shows the percentage of metrics used in the investigated articles. All the metrics commonly used to assess the performance of models are summarized in follows:

**Accuracy (Acc)** **=** $\frac{TP+TN}{TP+TN+FP+FN}$
**Sensitivity (Sen)** **=** **true positive rate (TPR)** **=** **recall (Re)** **=** $\frac{TP}{TP+FN}$
**Specificity (Spec)** **=** $\frac{TN}{TN+FP}$
**Precision (Pr)** **=** **positive predictive value (PPV)** **=** $\frac{TP}{TP\;+F}$
**False-positive rate (FPR)** **=** $\frac{FP}{TN+FP}$
**Negative predictive value (NPV)** **=** $\frac{TN}{TN+FN}$
**F1 Score (F1)** **=** $\frac{2TP}{2TP+FP+FN}$
**Classification Error Rate (CER%)** **=** $\frac{FP+FN}{TP+TN+FP+FN}$
**Receiver operating characteristic (ROC) curve and the area under the curve (AUC)**
**Youden's J statistic (J- Measure): J** **=** **Recall** **+** **Spec – 1** **=** $\frac{TP}{TP+FN}$ **+** $\frac{TN}{TN+FP}$ **−*1***

For classification in imbalanced data, Balanced Accuracy (BACC) and Matthew's Correlation Coefficient (MCC) are used:

**BACC** **=** $\frac{1}{2}\;$^*^ ***(***$\frac{TP}{TP+FP}\;$**+** $\frac{TN}{TN+FN}$***)***
**MCC** **=** $\frac{\left(TP^{*}TN\right)-\left(FP^{*}FN\right)}{\sqrt{\left(TP+FP\right)\left(TP+FN\right)\left(TN+FP\right)\left(TN+FN\right)}}$

where TP is a true positive, TN is a true negative, FP is a false positive, and FN is a false negative.

Performance in MI diagnosis is often measured by accuracy. Nonetheless, relying on accuracy alone might be misleading in the context of imbalanced data distributions, since it is easy to obtain a high accuracy score by simply classifying all observations as the majority class (e.g., the low prevalence of a disease). The AUC measures the ranking scores between predictions and targets, and we can identify the model that offers the best trade-off between specificity and sensitivity by using AUC (67). Medically speaking, AUC is more informative than accuracy. Other than the ROC plots and the AUC, the metrics mentioned above are all single-threshold measures and none of them can give an overview of performance when thresholds are varied.

In the F1 score, precision and recall are weighted by a harmonic mean. It is commonly used in binary classification problems. F1 score accounts for precision and recall of positive observations while accuracy reflects correctly classified positive and negative observations. It will make a huge difference for imbalanced problems since the model generally predicts true negatives. When both true negatives and true positives are equally important, then accuracy should be selected. If the dataset is heavily imbalanced or the positive class is mostly cared about, the F1 score is a better choice.

#### Data Splitting

In DL methods, data splitting is of great importance for performance evaluation. The model will likely fit the data to a maximum extent if all the original data are used to train the model while performing terribly for new data. Training-validation-testing data splitting is commonly used to prevent the over-fitting problem of the models. The three subsets perform different functions: the training set is used to train models and adjust parameters. The validation set evaluates whether the efficiency of model training goes in a bad direction and participated in the process of parameter tuning (hyperparameter), and the test set is used to evaluate the generalization capability of models. The proportion of data splitting varies in different studies, and there may be no validation set in some studies. K-fold cross-validation (CV) is a dynamic validation technique that aids in mitigating the impacts of data partitioning. The dataset is separated into k folds in k-fold CV. The model is trained using K-1 folds, and its accuracy is evaluated using the remaining 1-fold. This procedure will be repeated k times, with the final model performance calculated by averaging the results. Figure 4C depicts the distribution of data splitting approaches in the investigated MI research.

In some research, the class-based and the patient-specific experiments are designed to explore intra-individual variability (AIV) and inter-individual variability (RIV), respectively. It comes down to two different ways of data splitting. AIV can be investigated when the same individual patient's ECG heartbeats simultaneously appeared in both the training and testing datasets, whereas a patient-independent evaluation paradigm needs to confirm no overlap of individual patient data between the two datasets. In all the pieces of literature that employed both intra- and inter-patient experiments simultaneously, we can see that the models deployed in an intra-patient experiment always have overestimated prediction, this is due to the data in the test dataset has been partly occurred in the training dataset. Training and testing with the same data results in overfitting that yield to overestimated performance of the algorithm. Therefore, this kind of excellent performance does not mean this model can classify and detect unseen things very well in the future. RIV is a significant challenge for automated diagnosis since the ECG beats of the patients showed different characteristics, so this type of data splitting which is employed in an inter-individual scheme accords with the real world more than that in an intra-individual one. The evaluation results of the model deployed in the inter-patient experiment would better represent the real performance in real-world applications since networks are trained using a dataset of recorded patient data but applied to new patients and obtain better generalizability.

### Pre-processing

Instead of relying on the complex steps of feature extraction, DL methods learn the intrinsic characteristics directly from raw or low-level processed data. Relatively little preprocessing such as resample, denoise, segmentation, and data balance are also necessary for some research. Resampling ECG data to obtain a consistent sampling rate is essential to maintain consistency. In this stage, the ECG beats are changed into the same periodic length for the DL models. ECG waveforms can be subsequently deformed by two major artifacts including high-frequency noise as well as baseline wander (BW) caused by breathing and patient movement (75). Wavelet transform methods performed well in eliminating the ubiquitous ECG noises. Daubechies wavelet 6(db6) mother wavelet basis function and Savitzky-Golay (SG) smoothing filter are commonly used to filter out high-frequency noise and correct baseline wanders. In Acharya et al. (76) and Liu et al. (77), the ECG signals with noise and without noise are both used for CNN models to compare the performance, and the results illustrated that the higher quality denoised ECG signals can improve the performance of models. However, in Zhang et al. (78), heartbeats with and without noise are adopted in contrast experiments to verify the model's robustness to noise, and the research results indicated that the experiment using ECG heartbeats with noise achieved better performance. The main reason for this anomaly is that some informative features will be missed while using wavelet to denoise since the noise domain has an unescapable overlap with the information domain. Heartbeats are segmented by distinctive points, notably fiducial R-point since R-wave has an extremely high amplitude and plainly visible peak. Pan Tompkins algorithm is the most widely used R-peak and QRS-wave detection method and modified Nagatomo's method was conducted to automatically update the algorithm of R-peak threshold in Sugimoto et al. (79). According to the position of R-peak, sliding windows are generated with the same number of heartbeats to control the size of ECG records which inputted into the model due to the length of ECG signals varies in datasets. Data imbalance occurs when the number of samples for different classes varies greatly in a classification task, and it is another common issue of influencing model performance since it almost exists in all datasets. Low-degree imbalanced data does not matter much whereas there is a great deal of highly imbalanced data in medical diagnostics of the clinical setting. Large data skew can affect the prediction results. Several approaches have been discussed in reviewed literature to best address the problem of imbalance. Rai and Chatterjee (80) proposed a Synthetic Minority Over-sampling Technique (SMOTE) to create synthetic samples for minor classes instead of copies. SMOTE is a representative algorithm of the oversampling method. Hammad et al. (81) and Dai et al. (82) added a new loss function called focal loss (FL) to address data imbalance and results in Tripathy et al. (83) indicated that FL increased MI detection accuracy by 9%. Cao et al. (84) chose Balanced Cross-Entropy, which is a modified version of the cross-entropy loss function. To forcibly strengthen the robustness of DL models, data augmentation is also used to deal with the scarce class data and to supplement the trained model with more diverse and representative data. Alghamdi et al. (85) investigated MI ECG segments with and without the augmentation technique in the proposed model, and results indicated that the model with data augmentation technique achieved better performance than that without one. Besides that, in Darmawahyuni et al. (86), researchers introduced two performance metrics Balanced Accuracy (BACC) and Matthew's Correlation Coefficient (MCC) to evaluate the performance in their proposed study which the imbalanced ratio reaches 4.57. Notably, the balanced data helps to improve the model performance, nevertheless, there is a gap between balanced distribution and real condition (87).

Furthermore, to solve the problems of amplitudes scaling and eliminating offset effect, some researchers centralized and normalized each heartbeat, respectively (43, 68, 84). Rare studies converted one-dimensional (1-D) ECG data into two-dimensional (2-D) images and treated them as computer vision tasks. In this research, signal preprocessing steps can be nearly avoided.

### Architectures

**Figure 5:**
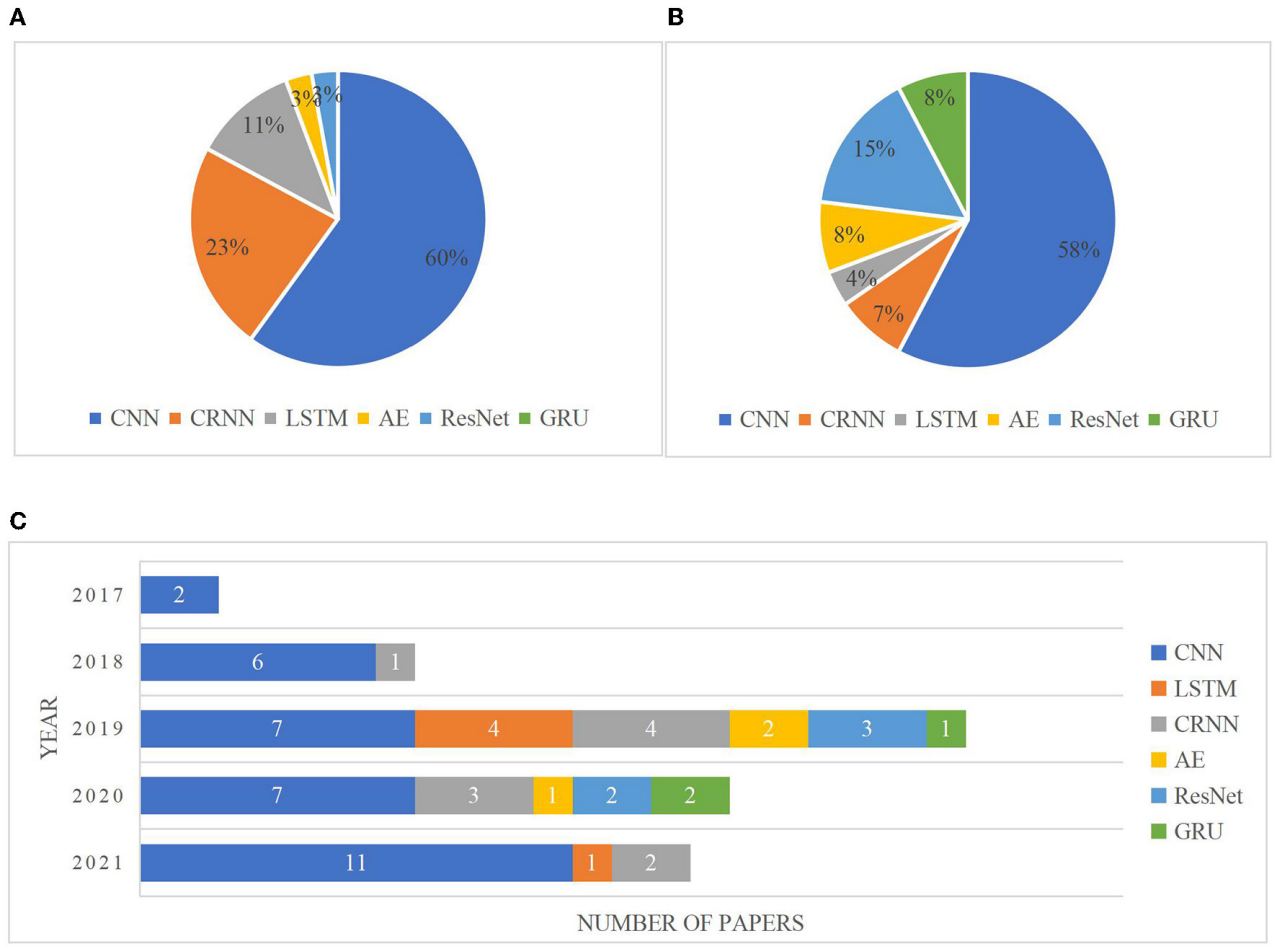
Percentages on different deep learning (DL)-based models for MI detection **(A)** and localization **(B)**. Convolutional neural network (CNN) is the most common technique used for both MI detection (with 60% contribution) and localization (with 58% contribution). Convolutional recurrent neural network (CRNN) (for MI detection) and ResNet (for MI localization) are the next most frequently used learning techniques in articles. Distribution of articles focused on each DL method in recent 5 years **(C)**. This figure shows the usage changes over the years of the six methods. Another four types of DL methods have been emerged for MI diagnoses from 2019 compared to that in 2018, while only CNN and CRNN were used in 2017 and 2018. CNN dominated the model categories in each year, and the proportion of CNN shows a gradual upward trend from 2019 to 2021 (33, 47, and 79 in 2019, 2020, and 2021, respectively).

A plethora of studies have utilized DL algorithms to vastly improve ECG waveform-based heart diseases classification. With a focus on MI classifications, aside from binary classifiers for detection of MI and non-MI, more studies have also accomplished multiple-class classification for localizing the distinct injury parts of the myocardium. According to a content review, six main types of DL models are concluded: CNN, LSTM, CRNN, ResNet, AE, and GRU. The popularity of each type of model in MI detection and localization is illustrated in Figures 5A,B, respectively. The usage changes over the years of the six methods are illustrated in Figure 5C. The popularity of the CNN model can be reflected in terms of research purposes and annual classification models. In the following sections, each of these models will be discussed in more detail.

#### CNN

Convolutional neural network (CNN) is not only well-known for its ability to complete computer vision tasks but also excellent in speech recognition, natural language processing, and signal analysis. CNN's basic structure is composed of a convolution layer followed by batch normalization layer, rectified linear activation function, pooling layer, and fully connected layer. The convolution process entails convolution of the input maps by kernels and then adding a bias to make the output maps. Multiple convolution layers and pooling layers are generally selected and set alternately, and the different convolution layers could extract different levels of features. The direct use of raw ECG data effectively alleviates the information loss which results from the process of handcraft feature extraction and selection. Table 4 lists the details of reviewed papers using the CNN model for MI detection and localization.

**Table 4:** Properties of some notable convolutional neural network (CNN)-based ECG MI detection and localization.

**References**	**Year**	**Data used**	**Architecture of CNN**	**Data splitting**	**Performance**		**Inter-/intra-** **patient analysis**
					**Detection**	**Localization**	
(76)	2017	PTB, lead II MI: 40,182 HC: 10,546	11-layer	10-fold CV	Acc = 95.22% PPV = 98.43% Sen = 95.49% Spec = 94.19%		Intra-patient analysis
(88, 89)*	2018	LTST, 2 or 3 leads ST: 266,275 Non-ST: 300,000 (Image samples)	Pretrained model: Google's Inception V3	80:10:10%	AUC = 89.6% F1 score = 89.2% Sen = 84.4% Spec = 84.9%		NR
(77)	2018	PTB, lead II MI: 13,577 HC: 3,135	13-layer CNN:	10-folds CV	Acc = 99.34% Sen = 99.79% Spec = 97.44%		NR
(87)	2018	PTB Lead V2, V3, V5, and aVL	Multi-lead CNN (ML-CNN)	5 folds CV	Acc = 96.00% Sen = 95.40% Spec = 97.37%		NR
(43)	2018	PTB, 12 leads MI: 48,690 HC: 10,646 (Six classes)	Multiple-Feature-Branch Convolutional Neural Network (MFB-CNN)	NR	**Intra-:** Acc = 99.95% Sen = 99.97% Spec = 99.90% **Inter-:** Acc = 98.79% Sen = 98.73% Spec = 99.35%	**Intra-:** Acc = 99.81% **Inter-:** Acc = 94.82%	Inter- and intra-patient analysis
(68)	2018	PTB Lead V1, V2, V3 MI: 41,087 HC: 18,640	Multi-Channel Lightweight Convolutional Neural Network (MCL-CNN)	NR	AUC = 95.50% Acc = 96.18% Sen = 93.67% Spec = 97.32%		NR
(69)	2018	PTB Lead II, III, and AVF IMI: 3,222 HC: 3,055	Three inception blocks	NR	Acc = 84.54% Sen = 85.33% Spec = 84.09% (Detection of IMI)		NR
(78)	2019	PTB lead II MI: 50,486 HC: 10,289	The lightweight CNN-like model (PCANet)	5 folds CV	**Intra-:** Acc = 99.49% Sen = 99.78% Spec = 98.08% **Inter-:** Acc = 93.17% Sen = 93.91% Spec = 89.20%		Inter- and intra-patient analysis
(90)	2019	PTB, 12 leads MI: 485,752 HC: 125,652	10-layer	70:15:15%		Overall Acc = 99.78%	NR
(91)	2019	PTB 12 leads	20 layers	10-fold CV 80% : 20%	Acc = 93.53% Sen = 93.71%		NR
(83)	2019	PTB, 12 leads MI: 14,274 HC: 2,826	Multichannel 1-D shallow CNN as classifier	70:30%		Acc = 99.84%(Seven classes)	NR
(92)	2019	Training: 483 MI, 474 non-MI Testing: 340 MI, 260 HC	CNN-based	7-fold CV	Acc = 94.73% Sen = 96.41% Spec = 95.94% F1-score = 93.79%		NR
(67)	2020	ECG and MRI 114 patients enrolled in the Kerckhoff Biomarker Registry	CNN with fully connected feedforward network	6-fold CV	AUC = 0.89 Acc = 78% Sen = 70% Spec = 84.3% (Detection of myocardial scar)		Intra- and inter-patient analysis
(93)	2020	PTB, 12 leads 148 MI 141 non-MI (records)	6 layers CNN	10 different training/ validation/test sets	F1-score = 83% Acc = 81%		NR
(94)	2020	PTB, Lead II MI: 368 HC: 80 (records)	DenseNet	Intra-patient: 10 folds CV	**Intra-:** Acc = 99.74% Sen = 98.67% Spec = 99.83% **Inter-:** Acc = 96.92% Sen = 89.18% Spec = 97.77%		Intra- and inter-patient analysis
(95)	2020	PTB, Lead II MI: 44214 (6362, 7 parts) HC: 6,157	Binary Convolutional Neural Network (BCNN)	10 folds CV	Acc = 90.29% Sen = 90.41% Spec = 90.16%		NR
(96)	2020	ECG-VIEW II MI: 201 Non-MI: 71 records	16 layers CNN	10 folds CV 80:10:10%	Acc = 91.1% Sen = 95% Spec = 80% Ppv = 93% F1 = 94%		NR
(85)	2020	PTB, Lead II MI: 80,364 HC: 21,092	Pre-trained VGG-Net VGG-MI1 VGG-MI2	10 folds CV 60:30:10%	Acc = 99.22% Sen = 99.15% Spec = 99.49% (VGG-MI2)		NR
(84)	2020	PTB, Leads v2, v3, v5, and aVL AMI: 38,536 HC: 18,640	Multi-Channel Lightweight Convolutional Neural Network (MCL-CNN)	10 folds CV 70:10:20%	Acc = 96.65% Sen = 94.3% Spec = 97.72%		NR
(81)	2021	PTB Lead II MI: 368 HC: 181	22-layer CNN model	5 folds CV	Acc = 98.84% Sen = 97.63 Ppv = 98.31% F1 score = 97.92%		NR
(56)	2021	GGH and GCI datasets, 12 leads MI: 218,101 HC: 105,032	CNN based feature extraction	NR	AUC = 94% (Prediction of occurrence- time in MI)		NR
(98)	2021	PTB Lead II	10 layers CNN	10 folds CV	Ppv = 99.58% Acc = 99.95% Sen = 99.95% Spec = 99.95%		NR
(71)	2021	PTB 3 VCG leads	7 layers deep CNN	NR	Sen = 99.88 % Spec = 99.65% Acc = 99.82%		NR
(82)	2021	PTB Lead II and 12 leads 3s: 17,972	11-layer CNN	10 folds CV	Acc = 99.84% Sen = 99.52% Spec = 99.95%		NR
(64)	2021	PTB-XL and private dataset 12 leads	10 NNs with same parameters but different initializations	NR		AUC: LMI: 0.969, IMI: 0.973 ASMI: 0.987, AMI: 0.961 ALMI: 0.996	NR
(14)	2021	PTB, 15 leads MI: 148 HC: 52	ConvNetQuake, (8 layers CNN)	NR	**Intra-:** Acc = 99.43% Sen = 99.40% Spec =99.45% PPV = 99.46% **Inter-:** Acc = 97.83%		Intra- and inter-patient analysis
(99)	2021	PTB MI: 312	12 lead-branch CNN	5 folds CV 10 folds CV	Acc = 95.76%	Acc = 61.82%	NR
(100)	2021	PTB MI: 50315 HC: 10593	DenseNet to obtain key features	10 folds CV		Acc = 99.87% Sen = 99.84% Spec = 99.98%	NR
(101)	2021	ESCDB lead L3	2-D CNN 7 layers	Dataset1 (DS1), DS2, and DS3.1 for intra- analysis DS3.2 for inter- analysis	Acc = 99.26% Sen = 97.8% Spec = 100%		Intra- and inter-patient analysis
(102)	2021	PTB VCG signals	Multi-channel multi-scale deep CNN	10-fold CV	Acc = 99.58% Sen = 99.18% Spec = 99.87%	Acc = 99.86%	NR
(17)	2021	PTB Detection: 4,136 MI Localization: 50,579 MI	MI-CNN (For detection) LL-CNN (For localization)	10-fold CV	Acc = 99.51% Sen = 99.71% Spec = 99.35%	Ppv = 99.25% Recall = 99.05% F1-score = 99.14%	NR

(*) This means the model performance in this article is better than another one. HC, healthy control; CV, cross-validation; NR, not reported; ALMI, Antero-lateral myocardial infarction; AMI, Anterior myocardial infarction; ASMI, Antero-septal myocardial infarction; LMI, Lateral myocardial infarction; IMI, Inferior myocardial infarction.

- Single-lead and multi-lead CNN. As shown in Section Datasets, the ECG data of lead II is commonly used for MI detection. In a highly cited paper (76), a deep 11-layer CNN was implemented using ECG beats of lead II for MI detection and obtained an average accuracy of 95.22 and 93.53% without noise and with noise, respectively. The data between leads is independent and the combination of ECG signals from multi-lead reflects heart features on multiple scales, so several methods are employed to ensure the data independence among different leads, such as the multi-channel technology (68, 84), the lead asymmetric pooling (LAP) and sub-2-D convolutional layers (103), independent feature branch (43), and inception blocks (69). In these multi-lead CNN models, correlations containing intra-beat, inter-beat, and inter-lead are captured. Each lead is in line with an independent channel or feature branch, and the feature map of each lead will be concatenated and integrated into fully connected layers for detection and localization. Through training samples, each lead can find which 1-D kernel is most suitable for them. In Cao et al. (84), the results show that the comparative single-channel CNN model forcibly used the same kernel in different lead data and performed worse than multi-channel CNN which can obtain better feature representation. It is well-demonstrated that the proposed multi-channel CNN model meets the independence of data. Instead of multi-lead ECG data, a multi-branch CNN was proposed by Hao et al. (92) by dividing ECG images evenly into 12 branches based on 12 leads and contributing to 12 separate networks.
- 1-D and 2-D CNN. In CNN-based MI detection methods, the two types 1-D CNN and 2-D CNN are commonly used. 1-D voltage amplitude ECG data which is represented as a time-series signal is input to the 1-D CNN model. Because of that using ECG images as inputs of the CNN model is more corresponds to the way that cardiologists diagnose and analyze abnormalities, an emerging number of researchers adopted 2-D CNN when ECG signals are treated as an image. The 2-D CNN models used for detection are always trained through a transfer-learning scheme including the pre-trained Google Inception V3, GoogLeNet, MnasNet, and VGG-Net rather than trained from the ground up, and that is the idea of retraining the existing models. The 2-D grayscale image models use the snapshots of 10 s or two consecutive R-peaks ECG images which are transformed from continuous ECG temporal dynamics. The feature encoding of time-frequency spectrograms is also performed as a computer vision task, and it contains multiscale waveform features and the spatial correlations between these features. Using the Gramian Angular Summation/Difference Fields (GASF/GADF) method to transform 1-D ECG signal into a 2-D image, Zhang et al. (78) developed a PCANet for detecting prominent features. In terms of this transformation, the loss of information that results from preprocessing such as noise removal can be prevented. Alghamdi et al. achieved the highest accuracy of 99.02 and 99.22% using VGG-MI1 and VGG-MI2 models, respectively, which are fine-tuned from pre-trained VGG-Net so that their proposed automatic diagnosis system obtains the ability to deploy in urban healthcare (85). Notably, the aforementioned sub-2-D convolutional layers employed in multi-lead feature analysis differ from conventional 2-D CNNs since, in traditional 2-D CNN, the convolutional calculations are performed in both row and column directions, which make it suitable for image recognition. However, this convolution operation undermines the independence of different leads because the ECG data of different leads (column direction) within the same time (row direction) will be convolved. Some intra-lead local changes in multi-lead ECG cannot be captured in conventional 2-D convolutions, and normal pooling based on a single pooling factor cannot efficiently utilize the multiscale features. These characteristics make the conventional 2-D CNN very unreasonable for multi-lead ECG classification.
- CNN for further detection. In the investigated studies based on CNN, MI detection and localization are two main objectives. However, there are also several extended research such as detection of myocardial scar (MS) (67) and prediction of occurrence-time in MI (56). MI results in myocyte necrosis, which is replaced by fibrous scars due to the myocardium being very weak at regeneration, leading to arrhythmias, heart failure, and even sudden death. MS is the indicator of IHD, and cardiac magnetic resonance imaging (CMI) with late Gd enhancement (LGE) is the standard method for diagnosing MS and assessing structural conditions of the myocardium (104) Gumpfer et al. (67) first try to adopt a deep learning model to predict MS based on ECG and additional clinical parameters and achieved better performance with an AUC score, sensitivity, specificity, and accuracy of 0.89, 70, 84.3, and 78%, respectively, on combine model (ECG data together with clinical parameters) than that on ECG model. The results suggest that further information on patients' clinical characteristics can improve the prediction outcomes. Compared to the DL method applied for ECG analysis, there is also large research focusing on automatic approaches for MS detection from MRI. In Moccia et al. (105), a 2D CNN model toward automatically segments MS for MI quantification based on ENet is proposed and the best-performing achieved 97% median accuracy and ~71% Dice coefficient over a 30-subject cohort, however, the computational training time is too long. The ECG interpretation for the detection of MS is insufficient and complex for healthcare professionals to diagnose MS so that ECG cannot be applied as an alternative for MRI currently, but the disadvantages of MRI are also obvious, such as expensiveness and limited usability for patients with severe renal impairment. Further exploration of automatic techniques for the detection of MS is needed.

#### LSTM

Based on recurrent neural networks, Hochreiter and Schmidhuber (106) first proposed the concept of LSTM. This network gained great popularity in speech recognition and machine translation in recent years. It is an expert in dealing with sequential data and could handle the vanishing or exploding gradient problem in the backward pass by entering a gate mechanism, which makes it more applicable than a recurrent neural network. The sequential characteristic of ECG data is in line with the dependency relationship between input and output data. The vanilla version of the LSTM unit is composed of four parts: input gate, memory block, forget gate, and output gate. Input and output gates are responsible for updating information across time-steps, while gate mechanisms control the input and output flow of information to the memory cells so that the LSTM can store or discard data selectively. Notably, researchers prefer to employ an LSTM-based method to detect MI not to localize myocardial infarct regions. Table 5 lists the details of papers using the LSTM model for MI diagnosis. Darmawahyuni et al. (86) compared the performance of LSTM with that of other two sequential model classifier standard RNN and GRU within different data splitting and results indicated that a simple LSTM network with 90:10% for training and testing set presented the best specificity of 97.97% for MI detection than RNN and GRU. The bidirectional LSTM (Bi-LSTM) network is a variant of LSTM. In Zhang and Li (107), the proposed Bi-LSTM model provided the input sequence in both forward and backward ways, which simultaneously capture past and future information, and the heartbeat-attention mechanism was incorporated to improve the 2% accuracy of MI detection and reached 94.77%. In Zhang et al. (108), researchers used 8 leads ECG data to detect AMI and IMI and achieved the highest accuracy of 99.91%.

**Table 5:** Properties of some notable long short-term memory (LSTM)-based ECG MI detection.

**References**	**Year**	**Data used**	**Architecture of LSTM**	**Data splitting**	**MI detection**					**Inter-/intra-** **patient analysis**
					**Acc (%)**	**Sen (%)**	**Spec (%)**	**Pr (%)**	**F1 score**	
(108)	2019	PTB, 8 leads 54,753 heartbeats	LSTM	90:10%	99.91					NR
(107)	2019	PTB, 12 leads MI: 369 HC: 79 (records)	Bi-LSTM	70:30%	94.77	95.58	90.48			NR
(109)	2019	PTB MI: 10,144 HC: 2,215	Standard RNN 1,2,3 hidden LSTM layers	80:20%		91		91	0.90	NR
(86)	2019	PTB 15 leads	RNN LSTM GRU	90:10%	97.56	98.49	97.97	95.67	96.32%	NR
(110)	2021	PTB, Lead II MI: 50,732 HC: 10,123	3 layers LSTM	10 folds CV	89.56	91.88	80.81			Inter- and intra-patient analysis

#### CRNN

A CRNN is the combination of two parts: CNN and RNN. As GRU, LSTM, Bi-LSTM, and Bi-GRU are all variants of RNN, CRNN comes in various forms such as CNN-LSTM (90, 100, 111–115), CNN-BiLSTM (116, 117), and CNN-BiGRU (15). Heart rate variability (HRV) is a measure of the amount of variation in the R-R interval from beat to beat (118). Some CNN-based methods just classified single heartbeat or ECG segments so that the beat-to-beat HRV cannot be utilized by them, whereas LSTM is well-suited to process heartbeat sequences and can analyze the beat-to-beat variations of the ECG morphology after the convolutional layers. Convolution networks extract spatial features and temporal properties are acquired through LSTM. According to the characteristics of the two networks, some researchers prefer to combine CNN with LSTM to create hybrid models. Theoretically, the combination of the two-deep learning techniques can capture any subtle morphological changes as well as beat-beat HRV and makes it excellent applicability in MI detection and localization. It is adopted by replacing one fully connected layer of the CNN classifier with an LSTM layer to create the CNN-LSTM classifier in Lui and Chow (119), and the technique of stack encoding is also added into the hybrid model to exhibit improved performance. Based on MFB-CNN in Liu et al. (43, 116) added a Bi-LSTM into a single model to abstract all 12 feature branches in total, and the model with the Bi-LSTM module can achieve better performance than the one without Bi-LSTM, notably, this is possible because Bi-LSTM is efficient in logical dependencies. Similarly, a shallow 1D CNN and a Bi-LSTM layer (117) were used to classify MI subjects from 21 temporal features which were collected from the 12-lead data. In Tadesse et al. (97), a 2D spectral-based CNN which enables cross-domain transfer learning from pre-trained GoogLeNet was used to encode frequency-time characteristics, and the corresponding spectral dense features are then fed into LSTM-based longitudinal modeling. Just one researcher hybrid the CNN with BiGRU (15), a novel MLA-CNN-BiGRU framework with multi-lead attention mechanism integrated for automatic MI detection and localization is employed, satisfactory performance was obtained in MI detection under intra- and inter-patient experiments but the MI location under inter-patient scheme needs further improvement. Table 6 lists the specifications of investigated papers using the CRNN model for MI diagnosis.

**Table 6:** Properties of some notable convolutional recurrent neural network (CRNN)-based ECG MI detection and localization.

**References**	**Year**	**Data used**	**Architecture of CRNN**	**Data splitting**	**Performanc**		**Inter-/intra-patient analysis**
					**Detection**	**Localization**	
(119)	2018	PTB and AF-Challenge, lead I 368 MI, 80 HC	CNN-LSTM stacking decoding classifier	10-fold CV	Sen = 92.4%, Spec = 97.7% Pr = 97.2%, F1 score = 94.6%		NR
(120)	2019	PTB, lead I 148 MI, 52 HC	16-layer CNN-LSTM	10-fold CV	Acc = 95.4%, Sen = 98.2% Spec = 86.5%, F1 score = 96.8%		NR
(121)	2019	PTB, Lead II Total 150,268	16-layer CNN-LSTM	10-fold CV	Acc = 98.51%, Sen = 99.30% Spec = 97.89%, Pr = 97.33%		NR
(116)	2019	PTB, 12 leads MI: 148 HC: 52 (368 records)	CNN combined with Bidirectional LSTM	5-fold CV		**Intra-:** Acc = 99.90%, Sen = 99.97% Spec = 99.54%, Pr = 99.91% **Inter-:** Acc = 93.08%, Sen = 94.42% Spec = 86.29%, Pr = 97.21%	Intra- and inter- patient analysis
(122)	2019	PTB, 12 leads	Multiple 1-D convolution layers and LSTM layers		Acc = 83% Sen = 79% Spec = 87%		NR
(97)	2020	PTB, 12 leads 148 MI, 52 HC	CNN features and LSTM-based network	5-fold CV	AUROC = 94%		NR
(123)	2020	PTB 15 leads	Enhanced Deep Neural Network (EDN)		CNN:84.95% LSTM: 85.23% EDN:88.89%		NR
(44)	2020	PTB 12 leads 632,940 MI 127,188 HC	2-D CNN and bidirectional gated recurrent unit (BiGRU) framework (MLA-CNN-BiGRU)	5-fold CV	**Intra-:** Acc = 99.93% Sen = 99.99%, Spec = 99.63% **inter-:** Acc = 96.50% Sen = 97.10%, Spec = 93.34%	**Intra-:** Acc = 99.11% Sen = 99.02%, Spec = 99.10% **inter-:** Acc = 62.94% Sen = 63.97%, Spec = 63.00%	Intra- and inter-patient analysis
(117)	2021	PTB 12 leads 80 HC 70 non-MI 367 MI records	Combination of only a shallow 1D CNN layer and 1 bi-LSTM layer	5-fold CV	Acc = 99.246% Sen = 99.25% Spec = 99.62% F1 = 98.86%		Intra-patient
(80)	2021	PTB and MIT Lead-II 123,998 beats	23 layers hybrid model 21 layers CNN	NR	Acc = 99.89% Spec = 99.77% F1 = 99.64%		NR

#### AE

Autoencoder (AE) was introduced as early as 1994, and it is another form of artificial neural network (ANN) for reducing dimensionality. The extended capability of this model is strong, and it can be applied to data dimension reduction, feature extraction, and data visualization analysis (79). It contains an encoder and decoder. The coding process involves converting the input vector to a hidden representation and then returning it to its original form in the decoding process. The efficiency of the learning process can be improved because the input vector is transformed into a lower-dimensional representation in the coding process. Staked Sparse Autoencoders (SAEs) (74) and convolutional autoencoder (CAE) (78) are both variants of an autoencoder and are used for feature extraction. Table 7 lists the properties of some AE models for MI classification. Zhang et al. (74) structured shallow SAEs which were trained in unsupervised learning to extract discriminative features, nevertheless, the classification was completed by the shallow classifier TreeBagger. Sugimoto et al. (79) constructed a CAE-based model to learn the temporal features of normal beats and calculate the deviation from the normal waveform for the input signals. Finally, the KNN classifier was employed to categorize the error vectors into one of 11 classes. In Zhang et al. (78) and Cho et al. (65) proposed a variational autoencoder (VAE) to reconstruct a precordial 6-lead ECG using a limb 6-lead ECG. The encoder and decoder comprised 6 CNN layers each and were connected by a 1-D dense layer. The DL-based algorithm with VAE is one novel point of this retrospective research, and it outperformed conventional DL methods for interpreting MI or STEMI using 6-lead ECG.

**Table 7:** Properties of some notable autoencoder (AE)-based ECG MI detection and localization.

**References**	**Year**	**Data used**	**Architecture**	**Data splitting**	**MI detection**				**MI localization**			
					**Acc (%)**	**Sen (%)**	**Spec (%)**	**AUC**	**Nc**	**Acc (%)**	**Sen (%)**	**Spec (%)**
(79)	2019	PTB, 12 leads MI: 295,344 HC: 58,296	CAE KNN	10 folds CV	99.87	99.91	99.59					
(74)	2019	PTB lead II 50,336 MI 10,588 HC	**Feature extraction:** SAE **Classify:** Tree Bagger	10 folds CV	99.90	99.98	99.52		11	98.88	99.95	99.87
(65)	2020	12 leads and 6 limb leads	CNN + AE	NA	STEMI MI	89.2 80	92.0 81.8	0.974 0.880				

*CAE, convolutional autoencoder; SAE, staked sparse autoencoder*.

#### ResNet

Deep residual networks were originally conceived for visual recognition tasks to overcome the degradation phenomenon that occurs in deeper networks with the network depth increasing. In 2015, ResNet was introduced by He et al. on the ILSVRC & COCO 2015 competitions and achieved excellent performance in image detection and classification tasks which greatly exceeded the level of other approaches in the previous years, consequently winning 1st place on ImageNet detection, ImageNet localization, COCO detection, and COCO segmentation in ILSVRC (124). The obvious characteristic of ResNet is the considerable depth which enables the network to have an extremely strong ability of representation. Recently, the generic ResNet framework has been applicable in time series classification tasks. Table 8 lists the details of notable papers using the ResNet model for MI classifications. The existing residual skip CNN was trained to detect MI and improved 4% in the accuracy compared with benchmark performance in Gopika et al. (125). In Strodthoff's approach, the two variants of CNN including fully convolutional architectures and ResNet-inspired architectures with skip-connections were adopted to distinguish AMI and IMI, and both architectures showed similar performance when applied to ECG data with multiple leads. In this research, the ECG signal matrix was the form of input and directly fed into the first 2-D convolutional layer of the ResNet (126). Differently, in Jafarian et al. (39), a single 1-D convolutional layer of each ECG lead was treated as a front-end to extract a pseudo-time-frequency representation and summarized as multi-lead discriminative features which are then input the first 2-D convolutional layer, and then three residual blocks were followed. The performance results showed that there is just one miss-classified MI in the five-folds cross-validation so that the average accuracy, sensitivity, and specificity of MI detection and localization reached almost 100%, respectively. Similarly, Han and Shi (127) developed a unique multi-lead ResNet with three residual blocks and feature fusion using ECG recordings from 12 leads to detect and localize 5 types of MI. Following three residual blocks, the global average pooling (GAP) layer and dropout are applied to improve generalizability. Furthermore, experiments under intra- and inter-patient schemes were both implemented and achieved an accuracy of 99.92 and 95.49% for the two schemes, respectively. Attractively, Wang et al. (128) proposed a multi-lead ensemble neural network (MENN) with three sub-networks and generated 15 and 12 sub-networks for AMI and IMI detection, respectively. The architecture of Net1 includes two residual blocks with reference in Xie et al. (129) to extract features, and using ResNeXt, Net3 realized a multi-branch residual network by adding group operations to the convolutional operation of the residual blocks. Furthermore, Net2 used two inception blocks. The three sub-networks extracted ECG features in different levels and the ensemble of three nets had superior performance on detection of AMI and IMI.

**Table 8:** Properties of some notable ResNet-based ECG MI detection and localization.

**References**	**Year**	**Data used**	**Model**	**Data splitting**	**Performance**		**Inter-/intra-** **patient analysis**
					**Detection**	**Localization**	
(125)	2019	Trained: 12,952 Tested: 1,600 (heartbeats)	Deep residual skip CNN		Acc = 99.3% Pr = 0.99% Sen = 99%, F1 = 0.99		NR
(126)	2019	PTB, 12 leads MI: 127 HC: 52 (records)	Fully connected and ResNet LSTM	10-fold CV	Sen = 93.3% Spec = 89.7%		NR
(128)	2019	PTB AMI: 25,539 IMI: 25,181 HC: 10,354	Multi-lead ensemble neural network (MENN)	5-fold CV	**AMI:** Sen = 98.35% Spec = 97.49%, AUC = 97.92% **IMI:** Sen = 93.17% Spec = 92.02%, AUC = 92.60%		Inter-patient analysis
(39)	2020	PTB, 12 leads 5,968 segments	Feature extraction + shallow NN End-to-end CNN	5-fold CV 60:10:30%	Acc = 98.21%, Sen = 97.50% Spec = 98.01%	Acc = 99.99%, Sen = 100% Spec = 100% (Detection and localization)	NR
(127)	2020	PTB, 12 leads 17,212 MI 6945 HC	Multi-lead ResNet Fusion of features	5-fold CV	Intra-: Acc = 99.92% Sen = 99.98%, Spec = 99.77% Inter-: Acc = 95.49% Sen = 94.85%, Spec = 97.37%	Intra-: Acc = 99.72% Sen = 99.63%, Spec = 99.72% Inter-: Acc = 55.74% Sen = 47.58%, Spec = 55.37%	Intra- and inter-patient analysis

#### GRU

A Gated recurrent unit (GRU) is likewise intended to solve the vanishing gradient problem of RNN. GRU has fewer trainable parameters and lowers computational complexity than LSTM, making it an enhanced form of LSTM with a faster training process. The reset gate and the update gate are the two most significant gates. The update gate is a merge component of input and output gates in standard LSTM, and it focuses on the past information of the previous moment which should be kept. The reset gate controls the previous information that should be disregarded in the current hidden state. In Prabhakararao and Dandapat (130), the GRU is chosen as the basic unit of RNN, and two multi-lead diagnostic attention-based RNN (MLDA-RNN) models were proposed for the classification of three MI severity stages including early, acute, and chronic MI. When RNN encoding size and latent-space size are adjusted to 32 and 64, respectively, the proposed model achieved the best accuracy of 97.79%. Based on (130), three GRU models were designed with no attention, only intra-lead attention, and intra- and inter-lead attention (IIL) modules respectively in Prabhakararao and Dandapat (131). The intra-lead attention module concentrates on the most discriminative ECG characteristics of each lead to generate a lead-specific attentive representation (LSAR), whereas the inter-lead attention module integrates all 12 representations. The two modules are used for reducing intra- and inter-lead redundancies. An ablation model with IIL modules as well as patients' clinical features was proposed to detect MI and outperformed other existing models, showing an accuracy of 98.3%. Be analogous to Bi-LSTM, a basic Bi-GRU consists of backward- and forward-propagating GRU units, and it can utilize information of past and future time step. The Bi-GRU model was adopted on eight ECG leads to locate five classes of MI and obtain an overall accuracy of 99.84% in Zhang et al. (73), and such excellent performance enables it to be applied to the computer-aided diagnostic platform as a MI localization algorithm. Table 9 summarizes the properties of the GRU model for MI detection and localization.

**Table 9:** Properties of some notable GRU-based ECG MI detection and localization method.

**References**	**Year**	**Data used**	**Architecture**	**Data splitting**	**Performanc**		**Inter-patient/** **intra-patient**
					**Detection**	**Localization**	
(73)	2019	PTB, 8 leads 54,753 heartbeats	ML-BiGRU	90%:10%		Acc = 99.84%	NR
(130)	2020	**STAFF III:** 3,609 EMI **PTB:** 2,107 AMI, 3,618 CMI 1,902 non-MI, 3,024 HC	RNN encoding block	5-fold CV	Acc = 97.79%, Sen = 97.6% Spec = 99.43%, AUC = 0.98 F1 = 97.65		Inter-patient analysis

*ML-BiGRU, Multi-lead bidirectional gated recurrent unit neural network; EMI, early MI; AMI, acute MI; CMI, chronic MI*.

### Model Performance

**Figure 6:**
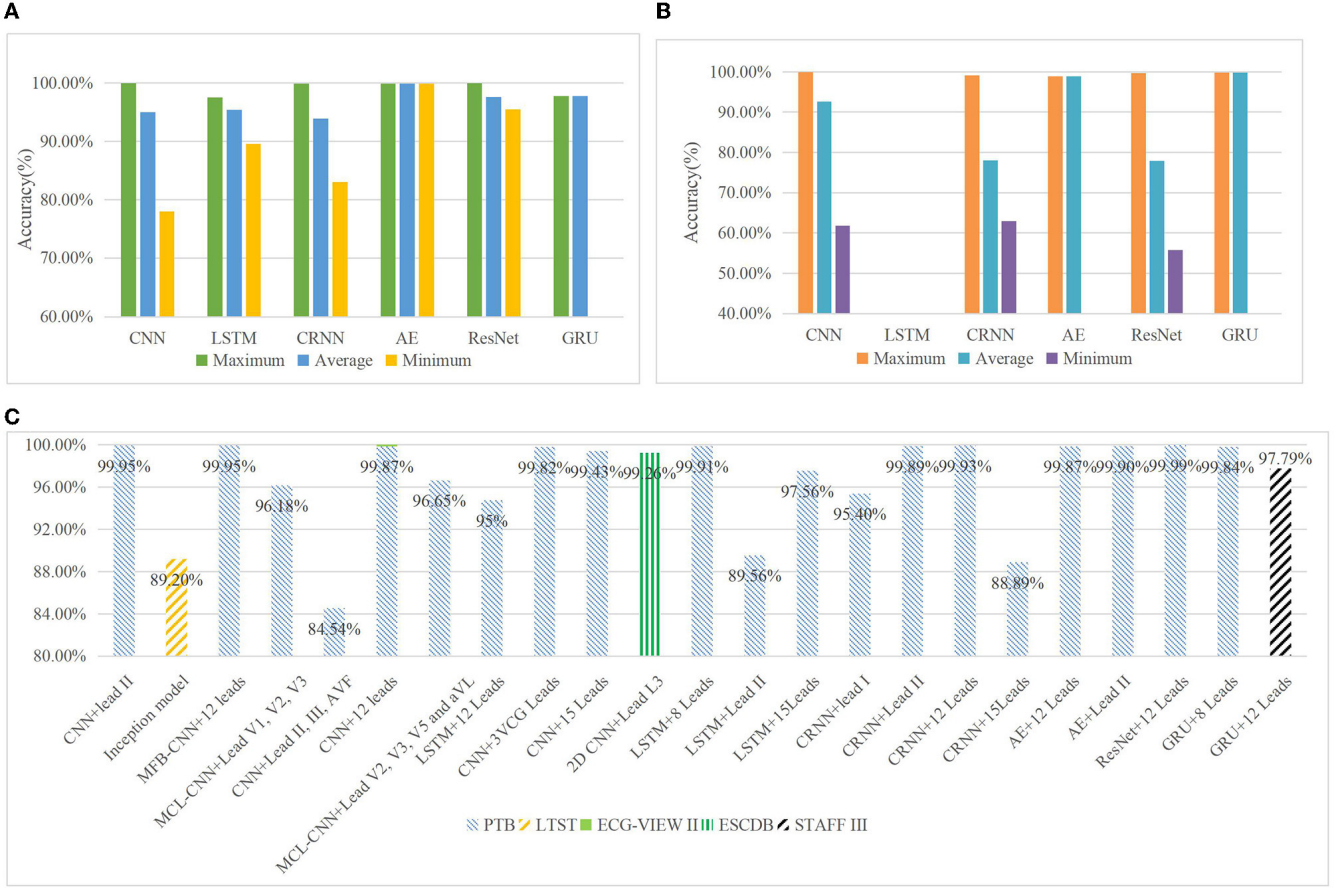
Maximum, average, and minimum accuracies of each DL technique for MI detection **(A)**. The reported highest accuracy for MI detection is 99.99% in the ResNet model, followed by CNN with an accuracy of 99.95%. The investigated papers with the reported highest accuracy for each model are all beyond 97%, and the average accuracy for each model is all beyond 93%. The reported minimum accuracy for MI detection appears in the CNN model with 78%. Maximum, average, and minimum accuracies of each DL technique for MI localization **(B)**. The reported highest accuracy for MI localization is 99.87% in the CNN model. There is no research focusing on applying the LSTM model to MI localization, and only one research for AE and GRU model, respectively, applied to MI localization. Therefore, the maximum and average accuracies of autoencoder (AE) and gated recurrent unit (GRU) models are the same, but the minimum is vacant. The minimum accuracies of the remaining three models are all below 65% in MI localization. Maximum accuracies of DL techniques which were trained on investigated ECG datasets and used the different number of leads **(C)**. Generally, the network that trained on 12 leads ECG data of PTB database has gained higher performance than that on smaller number leads of ECG data. ResNet model with 12 lead ECG data of PTB database achieved the highest accuracy 99.99% in MI localization. The usage of only lead II ECG data has also achieved good results in CNN, CRNN, and AE methods on the PTB database. The main reason is probably the three research with high performance that used only lead II ECG data are just for MI detection, and they cannot obtain matching results in MI localization.

Each original research covered in this review yielded encouraging diagnostic performances regarding the utility of DL in ECG interpretation. The advantages and disadvantages of the six different DL methods are reviewed in Table 10. To compare the most reported performance concerning the six different DL methods, Figures 6A,B display the maximum, average, and minimum accuracies of each DL technique for MI detection and localization, respectively. The maximum accuracies of DL techniques that were trained on reviewed ECG databases and used the different number of leads are summarized in Figure 6C. It provides us reference about selecting the appropriate data leads and datasets according to the purpose of the research since some smart bands and smartwatches record single-lead ECG and multi-lead Holter monitors. It is a little difficult and arbitrary to make a comparison of model performance among studies according to a single measurement due to variability that exists between different datasets and various metrics for measurements. However, some authors have conducted studies that compare different classification algorithms in their research, and using the same dataset makes the model performance more comparable. Table 11 summarizes the only six pairs of DL models reported in the investigated paper. This is also a flaw in MI studies. Studies comparing multiple DL techniques are expected to be conducted in future works.

**Table 10:** Advantages and disadvantages of the six different deep learning (DL) methods.

**Models**	**Advantages**	**Disadvantages**
CNN	The weight sharing strategy reduces the parameters that need to be trained	1. Amount of valuable information will be lost in the pooling layer 2. Poor interpretability
LSTM	1. Suitable for processing sequence signals 2. Overcoming the vanishing gradient problem occur on the timeline	The form of the LSTM neural network model is more complicated, and there are also problems like long training and prediction time
CRNN	It integrates the advantages of CNN and RNN (In MI research, recurrent layers are used to analyze the beat-to-beat variations of the ECG morphology after the convolutional layers)	High computational cost
ResNet	1. Training the network deeper 2. Fixed side effects of increased depth (degradation)3. Reducing the problem of information loss compared to CNN	The training time is longer
GRU	Making the structure simpler compared to LSTM but maintain the effect of LSTM	The performance of GRU is inferior to that of LSTM in the case of large datasets
AE	Performing feature dimension reduction, and facilitate data visualization analysis	The compression ability only applies to samples that similar to training samples

**Table 11:** Comparison of the methods that were reported in the articles.

**Model**	**Positive/Negative**	**MLP**	**CNN**	**LSTM**
CNN	+	*7.68% (85)		
CRNN	+	*12.73% (85)	*4.68% (85)	
	+	34.74% (15)	0.13% (68)	
	–		4.7% (150)	4.3% (150)
ResNet	+		1.8% (91)	2.23% (91)
	–			*4.56% (104)
GRU	+			1.20% (73)

The method in the corresponding row is more accurate than the method in the corresponding column when the number is positive and less accurate when the number is negative. (^*^) means the percentage is calculated by the precision of the two methods in the corresponding row and column. Only six pairs of model performance had been compared to each other in the investigated reviewed papers. Such comparisons are based on the same dataset. MLP: multiple layer perceptron.

## Discussion, Opportunities, and Open Issues

The number of articles about DL methods that were specifically used for the detection of MI has been largely increasing in recent 5 years. It also suggests that more and more researchers have explored the possibility of improving the performance of the diagnostic methods in DL. According to the detailed findings from the review, we also find some limitations about data quality, robustness, and interpretability of models. In addition to discussion about these limitations and challenges, new research opportunity regarding DL methods for detecting and localizing MI related to other current technologies such as the Internet of Health Things (IoHT) is also emerging. Besides that, open issues about privacy in healthcare data, the lack of annotated data of admissible quality, and the explainability of DL models are also being discussed.

### Limitations

#### Data Quality

First, the most mentioned limitation is the need for good quality and a large number of datasets. The sample size is smaller than 300 in five datasets investigated in this review, thus, sizable data is needed. PTB database has been the first choice in nearly 80% of research for MI detection and localization, although that includes using multiple different datasets simultaneously containing PTB. The usage of a singular dataset result leads to DL algorithms being too over-fitted to this dataset, hence the robustness of DL models cannot be guaranteed. Second, the demographic diversity of subjects which could affect the generalizability of DL models to ECG interpretation remains a challenge. From the used datasets in the reviewed papers, all the 13 datasets were collected from just a few continents, such as Europe (German, Slovenia, and European community), Asia (China, South Korea, and Pakistan), and North America (USA). When researchers in distant countries train their models using a uniform data set, the racial difference perhaps makes biased model predictions. It is important to focus on the problem of racial bias when considering AI into disease diagnosis since the diagnosis results to be biased by race are not expected. Several examples confirm the existence of this problem. For instance, Google's algorithm for diagnosis of diabetic retinopathy performed poorly in populations in India outside where the model had been developed (132) while Amazon recently created a facial recognition system, “Rekognition,” that was proficient at detecting lighter-skinned men but had trouble identifying darker-skinned women and men (133). Noseworthy et al. conducted a retrospective cohort analysis to assess the impact of race on the performance of their CNN model to detect low left ventricular ejection fraction (LVEF), but the prediction results did not correspond with the ethnic disparities. That suggests that the ECG features associated with LVEF rather than ECG are race-invariant. However, the effect of racial disparity on DL algorithms for other disease diagnoses should be further explored (134).

#### Performance

With the success of CNN architectures in these diverse domains such as image recognition and natural language process, researchers have applied them to time series analysis (111). In this review, CNN obtains the most popularity and gets as good performance as ResNet models in both MI detection and localization according to the previous statistical analysis. The analysis indicates that most researchers prefer to choose such a relatively old but proven reliable model and gradually improve this model to make the performance better and better in MI detection and localization. Then, it can be shown that the difference between the maximum accuracies of each DL technique for MI detection and localization is relatively small in Figures 6A,B. For instance, the difference between the maximum accuracies of CNN, CRNN, AE, and ResNet in MI detection, and the maximum accuracies of CNN and LSTM in MI localization are all <0.1%. Even the maximum accuracy of ResNet in MI detection is 0.04% higher than that of CNN, and the maximum accuracy of AE in MI localization is 0.01% higher than that of CNN. When these two tasks of detection and localization are considered simultaneously, CNN just wins a narrow victory. Finally, simply comparing the accuracy of different models in different experimental settings (data, model training, etc.) is a bit arbitrary, the lack of a unified standard for measuring and comparing is the main limitation. Another important problem is related to data imbalance. In the investigated studies, the ratio of different classes of data is generally below 1:4, it shows a slight imbalance and just a few researchers add techniques to deal with the problem. The AUC is an important statistical metric in the medical domain and is robust to imbalance distribution of positive and negative samples, and it can visualize this problem in plots, however, most researchers use accuracy to evaluate the model performance instead of AUC. To merely analyze the experimental data in the investigated papers, we found the size of the data seems to have an impact on the model performance. For instance, the accuracies of models trained on larger datasets (17, 43, 85, 90, 100) are higher than those on smaller datasets (67, 69). However, model performance is emphatically not influenced by a single factor. In most situations, more data is usually better, but more data does not always equivalent to a more accurate model since high bias models will not benefit from more training examples. Overfitting should also be considered.

#### Robustness

The robustness of a model refers to its ability to withstand perturbations or accurately classify the input data without adjusting its initial parameters. In brief, robustness can be understood as the tolerance of the model to data changes. In the reviewed studies, researchers adopted specific techniques to denoise, and the noise-free ECG signals were then input to networks. In real-world applications, the clinical setting is complicated and changeable, and ECG waveforms can be deformed by many external forces. The original ECG signal with noise will be the input for automatic devices. Therefore, adding some noise to the training samples is necessary and can improve the robustness of the models. Zhang et al. (78) has proved it. Zhang et al. adopt heartbeats with and without noise in contrast experiments to verify the model's robustness to noise, and the results indicate that the experiment using ECG heartbeats with noise indeed achieves 1% higher accuracy than that without noise. Notably, too much noise will make the training error larger. However, in Han et al. (135), show that even a model with very high accuracy could misclassify a normal sinus rhythm (NSR) recording as atrial fibrillation (AF) with high certainty by adding small perturbations to an ECG.

#### Explainability

The effect of “black box.” This limitation is also mentioned in Section Machine Learning for MI Diagnosis. The characteristic of learning features automatically from raw data rather than done manually makes the interpretability of DL approaches remains a general challenge, which is especially important for medical applications (112), because the mysterious processes may not be acceptable for medical professionals. There have been most research put forward exploratory studies for the application of interpretability methods in computer vision, whereas few applications covered time series ECG data. For example, attribution methods were investigated in Strodthoff and Strodthoff (126), which allows qualitative study if the DL algorithm uses similar features as human cardiologists. In some studies (67), explainable artificial intelligence (XAI) is still regarded as the future work to elucidate the mechanisms of DL models.

### Opportunities

Wearable devices have shown their potential for risk assessment, screening, early diagnosis, and patient management in some cardiovascular diseases such as hypertension, atrial fibrillation, heart failure, peripheral vascular disease, coronary artery disease, and so on (113). The MiCORE study in Marvel et al. (114) proved wearables' value of ECG monitoring in MI prevention. Two hundred patients with type I MI are enrolled, and the wearables include an Apple Watch and a Bluetooth BP cuff. Preliminary results showed a 43% lower likelihood of the first readmission within 30 days among patients in the Corrie Health Digital Platform (Corrie) than among participants in a historical comparison group. In the investigated studies, most researchers just build the DL models for MI detection and localization by using a fixed ECG dataset and few have deployed their developed models with great performance in wearable devices. Rashid et al. (95) designed a Binary Convolutional Neural Network (BCNN) for low-power and low-memory wearable devices with a single lead sensor, the microcontroller is similar to that of the SmartCardia device. The energy consumption evaluation shows that their model achieves 8 × and 12 × energy efficiency compared to other related works, so it is energy efficient and memory-saving for wearables. The current power consumption constraints of wearables might make researchers try to compress the DL models since the memory of digital devices is limited, and models using different lead data can be used in different devices.

Cloud computing offers some help to healthcare providers, such as hospitals and clinics, who need quick access to large storage facilities and computing. With cloud computing, healthcare providers can easily share healthcare data across regions, eliminating delays in patient care to realize smart medical monitoring. Cloud computing can be integrated into a platform for interaction and data sharing, especially for remote and personalized patient care in the medical (115). In medical diagnosis, various algorithms with promising performance have been proposed in large amounts of research, but they are rarely applied to an interactive platform and non-related professional and technical personnel have trouble using these models. Recently, an easy-to-operate platform LINDA which is designed for online medical image recognition has been proposed for common users without specific knowledge in the diagnostic area (136). The contribution of the LINDA system reflects in three aspects: firstly, it uses computational intelligence to recognize abnormalities instead of knowledge-based feature extraction. Second and most importantly, an application of image processing can be shared with other professionals to obtain more shared data for the improvement of model performance. Data privacy must be considered when involves data sharing. In the LINDA system, the hash code is used to prevent parameters from passing, and the number of requests to the prediction API is limited to reduce service attacks. In addition, the traffic assessment tools are also conducted. The third contribution is that it supports creating a large image database to self-improve. Similarly, in MI diagnosis, DL techniques can also be integrated into such a cloud platform to achieve data sharing and remote diagnosis, which further works are expected to explore.

### Open Issues

We are in the era of data explosion, the usage of smartphones, wearables, and IoT devices makes new data generate all the time. There is a lot of information that can be mined by DL algorithms in the massive data, but it also raises challenges about privacy. DL methods are data-driven methods. In the medical domain, the ethics related to patients' data that are being used for DL model prediction is of utmost importance. It makes sense to develop a solution that tries to find a balance between data utilization and privacy protection. The four stages of the life cycle model of big data in healthcare involve data collection, storage, sharing, and analysis process (137). Healthcare data subject involves individual patients, healthcare institutions, government, research institutions, and industry. Privacy leakage concerns can exist in every aspect and need to take the corresponding technical approaches to deal with. In data collection, data anonymity and differential privacy are the main technologies. Due to the size of sensors and wearables are getting smaller, lightweight, and less complex differential privacy algorithms are needed to adapt to such devices. Cloud computing is largely used to store big data in healthcare, and it is common to use encryption and auditing to ensure data confidentiality and integrity. The risks from data sharing could be effectively controlled through access control technology. In this way, novel technologies such as blockchain have been considered in encryption. In Khalid et al. (138), a decentralized authentication and access control mechanism is proposed and can apply to many scenarios. In Parah et al. (139), a novel high payload and reversible EHR embedding framework based on left data mapping (LDM), pixel repetition method (PRM), RC4 encryption, and checksum computation is proposed. In addition to technical support, policies, laws, and regulations are also necessary such as the European General Data Protection Regulation (GDPR). Hedlund et al. draw up a data sharing policy to provide guidelines for activities in the Analytic Imaging Diagnostics Arena (AIDA) (140). Privacy protection techniques in data analysis are especially important since lots of hidden information can be dug for only after data is analyzed. A research hotspot of model privacy is machine unlearning (141), and it is expected to realize model forgetting with less computational. To achieve smooth interoperability between the different parties, making data management is important.

In the medical domain, the input samples are always accompanied by various artifacts which lead to the lack of annotated data of admissible quality. However, the limited available input data in real-world practice make ML models may capture specifically these artifacts which are nothing to do with the diagnostic task (142). In terms of the problem of imperfect data quality, solutions including data augmentation techniques, transfer learning, and domain adaptation provide help. How to generate high-quality input samples instead of simply producing more training data through data augmentation has become the key issue. Recently, there are quite a few related studies, such as the classic Synthetic Minority Oversampling Technique (SMOTE), MixUp (ReMix), Hard Negative Mixing (143), and good-enough example extrapolation (144) all aim to improve the quality of resampling of small samples. The transfer learning emerges for solving the problem of insufficient annotation data, so it can be carried out under fast modeling, annotation deficiency, and small data modeling. However, the parametric transfer learning methods are time- and cost-consuming to tune, hence restricting the wide applicability in computational constraints of wearables devices. In Wang et al. (145), propose a first easy non-parametric transfer learning approach, the result of the extensibility experiment shows this easy approach can efficiently achieve good performance without requiring feature learning algorithms. Domain adaptation (DA) is an important part of transfer learning to solve the problem that data distribution between the source domain and target domain does not correspond with the independent and identically distributed (IID) condition. Gradient reversal layer (146) and generative adversarial network (GAN) (147) have been successfully applied in unsupervised domain adaptation.

In terms of input samples with artifacts mentioned before, another helpful solution is the interpretability of the models. The interpretability could enable it to notice abnormal predictions before being widely used as a diagnostic tool. The methods to achieve interpretability enable people with specific experience to have the maximum understanding of a specific model when given specific data and tasks. Based on the existing methods, we can divide the methods to achieve interpretability into three stages, that is, before, during, and after model training. Before model training, the objective of interpretability lies in data analysis, and what we're trying to do is learn about the data to the maximum extent. The methods used generally include data visualization and statistical analysis such as maximum mean discrepancy (MMD). In modeling, directly building interpretable models is a vital method. Linear regression models and specific neural networks with an explicit pooling structure are all self-explanatory models. Linear models and some of their variants have good explainability due to a very solid statistical foundation. Attention mechanisms are always adopted to provide insight into model prediction. In Samek et al. (148), four *post-hoc* explanation techniques have been summarized as follows: interpretable local surrogates, occlusion analysis, gradient-based techniques, and layerwise relevance propagation (LRP). A popular approach of local surrogates is the Locally Interpretable Model-agnostic Explanations (LIME) (149), which proposes to explain single predictions of any ML classifiers, and attribution method based on Shapley values which is a framework firstly proposed in game theory is a kind of occlusion analysis (150).

## Conclusion

Ischemic heart disease has been a major killer endangering human health. Myocardial ischemia leads to myocardial damage and necrosis, resulting in MI. Mild symptoms such as arrhythmias, ventricular premature, and ventricular block can be treated early but severe cases like cardiac arrest or even sudden death are catastrophic. Looking back on the news reported by the media in recent years, more and more young people, such as students, white-collar workers, and so on, are in a state of physical and mental fatigue for a long time due to the pressure of study and work, and unfortunately die suddenly. It suggests that this heart disease, which mainly occurs in the elderly, gradually tends to be younger. Therefore, achieving the early prevention of MI with long-term detection through ECG is vital to lifesaving. Based on the development of AI, big data, wearable devices, cloud healthcare, etc., automatic real-time monitoring of ECG data has become possible. In recent years, an emerging number of researchers engaged in the DL field, and DL methods have generally achieved outstanding performance in ECG interpretations. This review systematically summarizes the progress of DL methods for MI detection and localization, recaps some general limitations from the aspects of data, models, performance, and prospects the application of these DL technologies in clinical scenarios. It is hoped to provide some suggestions and references for researchers in related fields in model selection, the dataset used, and the construction of a cloud platform for real-time monitoring and diagnosis of ECG data. With the problem of data privacy always challenging, in the future, this monitoring paradigm could revolutionize cardiovascular care as soon as data security and other concerns are addressed. It is believed that more patients and sub-healthy patients can benefit from it and the mortality rate of cardiovascular diseases could decrease significantly.

## Author Contributions

PX and GC conceived of this work. PX collected and analyzed the data and wrote the draft of the manuscript. PX, SL, and GC reviewed and revised the manuscript.

## Funding

This work was supported by the Science and Technology Development Fund, Macau SAR (SKL-QRCM(UM)-2020-2022).

## Conflict of Interest

The authors declare that the research was conducted in the absence of any commercial or financial relationships that could be construed as a potential conflict of interest.

## Publisher's Note

All claims expressed in this article are solely those of the authors and do not necessarily represent those of their affiliated organizations, or those of the publisher, the editors and the reviewers. Any product that may be evaluated in this article, or claim that may be made by its manufacturer, is not guaranteed or endorsed by the publisher.
